# Postbiotic sequestration of micro- and nanoplastics: *in vitro* intestinal epithelial protection and proof of concept in the human mouth

**DOI:** 10.3389/fmicb.2026.1881786

**Published:** 2026-09-21

**Authors:** Eva A. Berkes, Oded Oron, Ana Wood, Phineas N. Monsul, Nicholas T. Monsul

**Affiliations:** Quorum Innovations Inc., Sarasota, FL, United States

**Keywords:** human microplastic exposure, chewing gum, *Limosilactobacillus fermentum*, microplastics, nanoplastics, postbiotic, microplastic sequestration, simulated digestion

## Abstract

Micro- and nanoplastics (MNPs) are ubiquitous dietary contaminants, yet practical strategies to reduce gastrointestinal exposure remain limited. We evaluated whether Qi601, a heat-inactivated *Limosilactobacillus fermentum* biofilm-derived postbiotic, could sequester fluorescent 100-nm polystyrene nanoplastics (PSNPs) and reduce their interaction with Caco-2 intestinal epithelial monolayers using sequential simulated digestion, protection, post-exposure rescue, and transfer assays. Consistent with particle sequestration, Qi601 produced approximately 92% depletion of free PSNP-associated fluorescence from the supernatant during sequential digestion. In protection assays, Qi601 reduced surface-associated and intracellular PSNP fluorescence by 65% and 67%, respectively, at 5 h, and by 41% and 33% at 24 h. In monolayers previously exposed to PSNPs for 24 h, Qi601 reduced surface-associated and intracellular PSNP fluorescence by 77% and 43%, respectively. Transfer of PSNP fluorescence from Qi601–PSNP agglomerates to Caco-2 cells remained ≤ 0.057%. Atomic force, scanning electron, and confocal microscopy demonstrated close physical association between Qi601 and PSNPs with predominantly extracellular localization of Qi601-associated PSNPs. In a qualitative human proof-of-concept chewing-gum experiment, microscopy of saliva showed heterogeneous, Nile Red-positive polymers within or closely associated with Qi601 agglomerates. Laser direct infrared (LDIR) chemical imaging showed that the number of rubber-class particles from the gum-plus-Qi601 saliva sample was 43% lower than from the gum-only sample (2,562 versus 4,529 particles; hit quality index ≥ 0.70); after procedural-control subtraction, the background-adjusted rubber particle count was approximately 91% lower (185 versus 2,152 particles; hit quality index ≥ 0.70). These findings establish proof of concept for postbiotic-mediated sequestration of dietary MNPs and support a model in which Qi601 limits their epithelial association and intracellular burden. To our knowledge, this represents the first human *in vivo* proof-of-concept study directly demonstrating physical association between an administered intervention designed to capture microplastics and microplastic particles. Further studies should define the molecular basis of Qi601–MNP interactions, characterize their gastrointestinal fate, and validate these findings in larger studies.

## 1 Introduction

Over the past century, global plastic production has grown rapidly from essentially zero to hundreds of millions of tons per year. More than half of all plastic ever produced has been manufactured since 2000, resulting in substantially higher baseline human exposure than in previous generations (Geyer et al., 2017). The durability of plastic results in long-term accumulation in the environment, where plastic waste progressively fragments into micro- and nanoplastics (MNPs) (Andrady et al., 2011; Hartmann et al., 2019).

The ubiquitous presence of MNPs in water, air, and food has raised growing concern about their potential ecological and human health impacts. Suggesting systemic exposure, MNPs have been detected in many human tissues, including blood, intestinal tract, kidney and brain (Schwabl et al., 2019; Leslie et al., 2022; Pironti et al., 2023; Nihart et al., 2025). Nanoplastics are of particular concern because their small size may render them able to cross biological membranes (Mattsson et al., 2017; Gigault et al., 2018; Xu et al., 2019).

Current approaches to reducing human MNP exposure are primarily environmental. Commonly recommended household, behavioral and dietary measures include avoiding heating food in plastic containers, use of natural fiber clothing, filtering drinking water and reducing indoor dust. Emerging studies have also examined biological strategies such as chitosan and wheat bran to bind or sequester ingested plastic particles, while microbiome-based approaches have been investigated for plastic adsorption, transformation, or degradation (Jang et al., 2024; Bornstein et al., 2025; Casella et al., 2025; Wang et al., 2026). Whether these approaches can reduce gastrointestinal MNP burden in humans remains unknown.

Probiotic bacteria and their metabolites have also recently been investigated as potential MNP-mitigation strategies. Live bacterial strains have been shown to adsorb MNPs, and studies in animal models have reported increased fecal elimination, reduced tissue retention, and improved intestinal barrier, inflammatory, or toxicity-related endpoints (Zhao et al., 2025; Teng et al., 2024; Demarquoy, 2025; Hwang et al., 2025; Shi et al., 2025; Lee et al., 2026). However, probiotic approaches to MNP mitigation pose several limitations, including variable survival during gastrointestinal transit, uncertain colonization or persistence in the host, shelf-stability constraints, susceptibility of probiotic organisms to MNP toxicity, and the theoretical possibility that live organisms could retain bound particles near the mucosal interface rather than promote their luminal clearance (Han et al., 2021; Wang and Zhong, 2024; Tao et al., 2025).

Unlike these previously described live probiotic approaches, the present study evaluates sequestration by a non-viable microbial preparation, thereby separating particle-plastic interaction from bacterial viability, colonization, persistence and metabolic activity. Because postbiotics are preparations of inanimate microorganisms and/or their components that confer a health benefit, they may offer advantages in this context by eliminating the need for microbial viability (Salminen et al., 2021). Although live probiotic strategies for MNP adsorption and toxicity mitigation are emerging, little is known about whether postbiotic microbial material can function as a luminal sequestration platform for MNPs under physiologically-relevant digestive conditions. The present study addressed this question using Qi601, a heat-inactivated biofilm-derived preparation of *Limosilactobacillus fermentum*, and evaluated its interactions with nanoplastics in experimental models relevant to the human gastrointestinal environment.

Accordingly, the present study combined simulated gastrointestinal digestion, intestinal epithelial cell models, complementary imaging modalities, and an exploratory human proof-of-concept experiment to evaluate postbiotic sequestration of MNPs (Figure 1). Because gastrointestinal transit can alter MNP aggregation, surface properties, biological interactions, and toxicity (Chen et al., 2026), fluorescent 100-nm PSNPs were evaluated in a sequential simulated human digestion model adapted from the INFOGEST protocol (Hubatsch et al., 2007; Brodkorb et al., 2019). The resulting digestates were subsequently evaluated in Caco-2 intestinal epithelial monolayers using complementary quantitative assays and multimodal imaging to examine Qi601–MNP interactions. Finally, a human proof-of-concept chewing-gum experiment using fluorescence microscopy and laser direct infrared (LDIR) spectroscopy was performed to evaluate the association of Qi601 with heterogeneous microplastics released during mastication. We hypothesized that Qi601 would physically associate with dietary MNPs under simulated digestive and physiologic oral conditions, retain them within postbiotic–plastic agglomerates, and thereby reduce epithelial interaction and intracellular particle burden.

**Figure 1:**
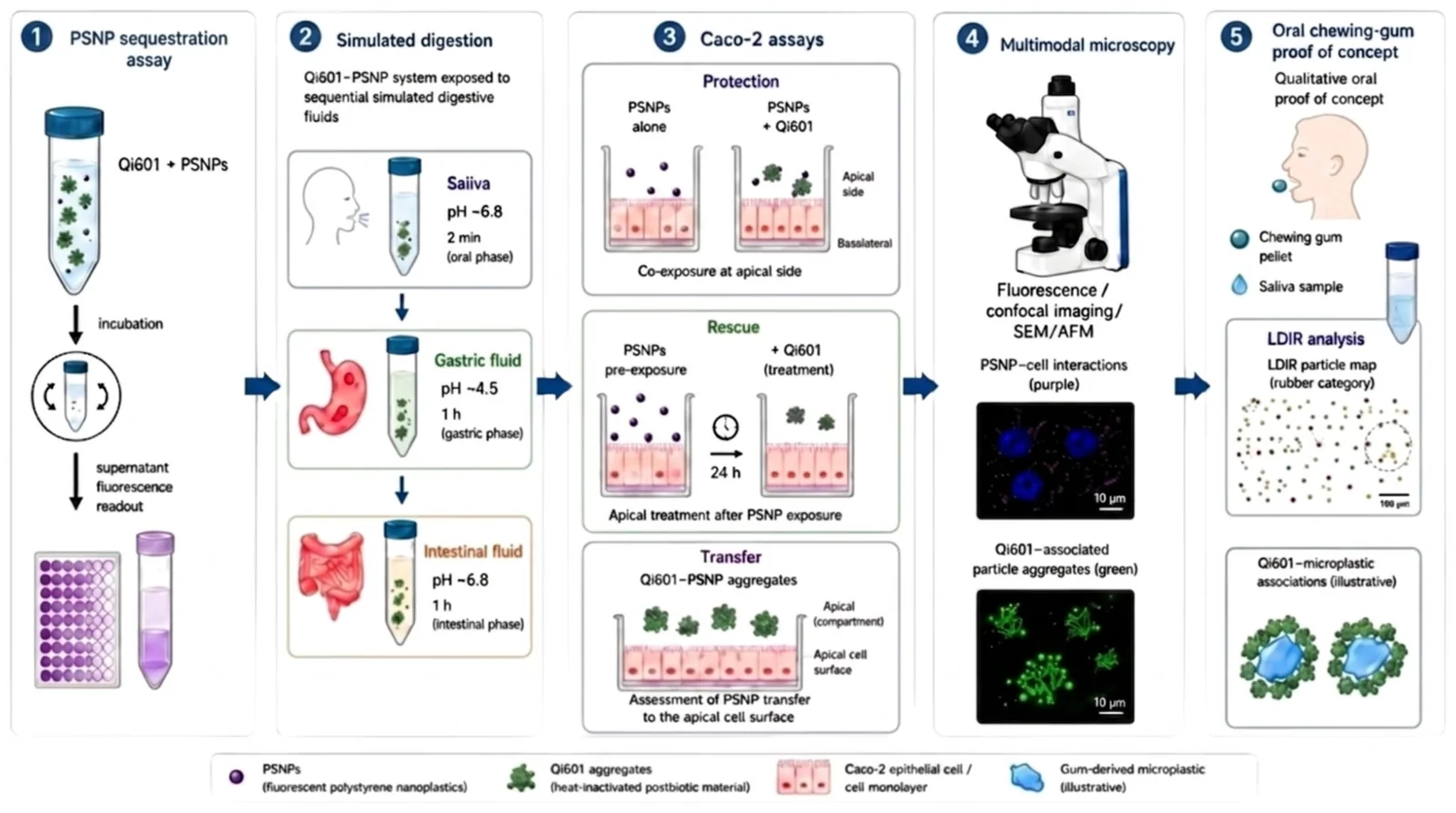
Experimental framework for evaluating Qi601 postbiotic-mediated sequestration of micro- and nanoplastics. Qi601 interactions with fluorescent polystyrene nanoplastics (PSNPs) were evaluated using a sequestration assay based on residual supernatant fluorescence. The Qi601–PSNP system was then subjected to sequential simulated salivary, gastric, and intestinal digestion. Caco-2 assays examined PSNP association with epithelial cells during co-exposure with Qi601, after PSNP pre-exposure followed by Qi601 treatment, and after exposure to preformed Qi601–PSNP aggregates to assess transfer to the apical cell surface. Fluorescence microscopy, confocal microscopy, scanning electron microscopy, and atomic force microscopy were used to examine PSNP–cell interactions and Qi601-associated particle aggregates. An oral chewing-gum proof-of-concept experiment was used to collect saliva samples for fluorescence microscopy and LDIR analysis of gum-derived microplastic particles. Created with BioRender.com.

Terminology: MNP denotes micro- and nanoplastics collectively; MP denotes microplastic; NP denotes nanoplastic; PSNP denotes polystyrene nanoplastic; PSMP denotes polystyrene microplastic; QPA denotes Qi601-PSNP agglomerates; DF denotes combined digestive-fluid mixture; DF/EM denotes DF diluted in complete Eagle's Minimum Essential Medium.

## 2 Materials and methods

### 2.1 Postbiotic preparation and characterization

The postbiotic Qi601 material was provided by Quorum Innovations Inc (Sarasota, FL). The strain in this study, *Limosilactobacillus fermentum* LfQi6, has been determined as Generally Recognized as Safe (GRAS) for its intended use (U.S. Food and Drug Administration, 2022). The strain was cultivated as a biofilm culture, centrifuged, washed, heat-inactivated and lyophilized using proprietary methodology in a cGMP-registered facility (Berkes et al., 2020). The resulting food-grade postbiotic preparation (“Qi601”) consisted of non-viable intact *L. fermentum* LfQi6 cells derived from biofilm culture. Representative SEM images of the final lyophilized preparation demonstrated intact rod-shaped cells organized into heterogeneous multicellular aggregates. AFM and fluorescence microscopy further confirmed the aggregate morphology of the final preparation. All experiments were performed using a single commercial-scale production lot of Qi601.

### 2.2 Evaluation of Qi601 dose-dependent *in vitro* PSNP sequestration

Qi601 dose-dependent sequestration of fluorescent PSNPs was evaluated in a supernatant-depletion assay. Fluorescent 100-nm polystyrene nanoplastics were selected as a well-characterized model particle to facilitate fluorescence-based quantification under controlled experimental conditions. For the purposes of this study, the term sequestration refers to removal of free MNPs from suspension through physical association with Qi601, irrespective of the specific underlying mechanism. Previous probiotic studies have generally described microbial–plastic interactions as adsorption. In the present study, the broader term sequestration is used because the experimental methods do not distinguish the relative contributions of surface adsorption, agglomeration, and entrapment. Accordingly, percentage sequestration values derived from this assay represent the reduction in PSNP fluorescence remaining in the supernatant relative to PSNP-only controls, rather than direct particle enumeration in the Qi601-associated pellet.

Qi601 at doses of 0.5–10 mg was suspended in 900 μL phosphate-buffered saline (PBS). A 1:1 (v/v) dilution of the manufacturer-supplied PSNP stock was prepared in PBS, and 10 μL of the diluted suspension was added to each sample (Fluoresbrite™ YG Microspheres, 0.1 μm; Polysciences, Inc., Warrington, PA, USA; catalog no. 07150). PSNP-only controls and each Qi601 treatment condition comprised three independent biological replicates. Each biological replicate was measured in technical triplicate. Controls included PSNP-only, Qi601-only, and PBS-only. PSNP-only controls were used to account for background particle sedimentation and self-agglomeration in the absence of Qi601.

Samples were incubated for 1 h at 37 °C with shaking at 250 rpm and then centrifuged at 1,700 × g for 10 min. Supernatants were transferred to black 96-well plates, and fluorescence was measured using a multi-mode microplate reader (Synergy LX, Agilent BioTek Instruments, Santa Clara, CA, USA) with excitation/emission wavelengths of 485/528 nm and a gain setting of 50. Fluorescence was corrected using the corresponding PBS and Qi601-only controls. The percentage reduction in PSNP fluorescence remaining in the supernatant was calculated relative to the PSNP-only control as follows:

% Reduction in supernatant PSNP fluorescence = [($\frac{{\text{F}}_{\text{PSNP}}-{\text{F}}_{\text{PSNP}+\text{Q}\text{i}601}}{{\text{F}}_{\text{PSNP}}}$)] x 100

where *F*_*PSNP*_ is the background-corrected fluorescence of the PSNP-only supernatant and *F*_*PSNP* + *Qi*601_ is the background-corrected fluorescence of the corresponding PSNP-plus-Qi601 supernatant. The lowest Qi601 dose producing near-maximal reduction in supernatant PSNP fluorescence (3 mg) was selected for subsequent experiments.

### 2.3 Using simulated digestion to evaluate nanoplastic interactions with Qi601

A sequential *in vitro* digestion model adapted from INFOGEST was used to evaluate interactions between Qi601 and PSNPs under simulated oral, gastric, and intestinal conditions. The standard INFOGEST protocol was developed primarily for food digestion and therefore was adapted for evaluation of Qi601–MNP interactions (Brodkorb et al., 2019). Retained features included sequential oral, gastric, and intestinal phases; phase-appropriate digestive fluids and pH conditions; pepsin-containing gastric fluid; bile salt-containing intestinal fluid; and digestion-phase exposure periods. Modifications included the use of commercially prepared digestive fluids, fed-state simulated gastric fluid to model postprandial dietary MNP exposure, reduced assay volumes, and orbital shaking to maintain particle suspension. Pancreatin was intentionally omitted because it could promote protein-corona formation and alter particle aggregation, confounding interpretation of Qi601–plastic interactions. Fluorescent 100-nm PSNPs were subjected to sequential simulated digestion in the presence or absence of Qi601. For the sequential simulated-digestion experiment, PSNP-only controls comprised three independent biological replicates and the Qi601 treatment condition comprised nine independent biological replicates. Each biological replicate comprised technical triplicates.

Qi601 was prepared as before and 100 μL was added to each designated tube. A 1:1 (v/v) dilution of the manufacturer-supplied PSNP stock was prepared in PBS, and 10 μL of the diluted suspension was added to each PSNP-containing sample. For the oral phase, samples were combined with artificial saliva (200 μL AS; Pickering Laboratories, Inc., Mountain View, CA, USA; pH 6.8; catalog no. 1700-0304) and incubated for 3 min at room temperature. Fed-state simulated gastric fluid (800 μL; FeSSGF; BioChemazone, Inc., Leduc, Alberta, Canada; pH 4.5; catalog no. BZ415) was then added, and samples were incubated at 37 °C for 1 h with orbital shaking at 250 rpm. Artificial intestinal fluid (800 μL; AIF; BioChemazone, Inc., Leduc, Alberta, Canada; pH 6.8; catalog no. BZ176) was subsequently added, followed by incubation for an additional 1 h at 37 °C with orbital shaking at 250 rpm.

In addition to the sequential digestion assay, parallel single-fluid assays were conducted in AS, FeSSGF, and AIF to evaluate Qi601–PSNP association in each digestive fluid individually. Corresponding PSNP-only, Qi601-only, and fluid-only controls were included.

Digested samples were centrifuged as previously described to pellet Qi601-associated material. Supernatants were collected without disturbing the pellet, transferred to fresh tubes, and plated in black 96-well plates in technical triplicate. Fluorescence was measured as described above, and background-corrected fluorescence was used to calculate PSNP removal from the supernatant relative to PSNP-only controls. Digestate pellets were frozen at −20 °C for subsequent Caco-2 experiments.

### 2.4 Generation of *in vitro* human intestinal epithelial cell model

Caco-2 cells (ATCC^®^ HTB-37™; American Type Culture Collection, Manassas, VA, USA; RRID: CVCL_0025HTB-37™) were used for *in vitro* PSNP uptake experiments. Caco-2 is a human colorectal adenocarcinoma-derived epithelial cell line commonly used as an intestinal epithelial model. Cells were maintained in T-75 culture flasks (Corning Inc., Corning, NY, USA; catalog no. 430641) in complete Eagle's Minimum Essential Medium (EMEM; American Type Culture Collection, Manassas, VA, USA; catalog no. 30-2003) supplemented with 20% v/v fetal bovine serum (FBS; Gibco, Thermo Fisher Scientific, MA, USA; catalog no. A5256701) and 1% penicillin-streptomycin solution, 10,000 U/mL (Gibco, Thermo Fisher Scientific; MA, USA; catalog no. 15140122).

Cells were incubated at 37 °C in a humidified atmosphere containing 5% CO_2_. Medium was refreshed every 2 days until cells reached approximately 50% confluence, after which medium was refreshed daily until cells reached 80–95% confluence. For subculturing and experimental seeding, cells were trypsinized at 37 °C with 0.25% trypsin/2.21 mM EDTA (Corning Inc., Corning, NY, USA; catalog no. 25-053-CI) and neutralized with an equal volume of trypsin neutralizer (Gibco, Thermo Fisher Scientific, Waltham, MA, USA; catalog no. R002100). The cell suspension was centrifuged at 400 × g for 10 min, and the cell pellet was resuspended in 1–2 mL complete medium.

For experiments, cells were seeded into flat-bottom 24- and 96-well plates (Costar, Corning Inc., Corning, NY, USA; catalog nos. 3524 and 3595, respectively) at 2 × 10^4^ cells/well. Before counting and seeding, cell suspensions were mechanically triturated by passage through a 25-gauge needle (Becton, Dickinson and Company, Franklin Lakes, NJ, USA; catalog no. 305125) to minimize cell aggregation and spheroid formation. Experiments were performed once cells reached approximately 85% confluence.

### 2.5 Assessment of Caco-2 monolayer tolerance to simulated digestive fluids

To establish Caco-2 exposure conditions compatible with the adapted digestion model, experiments were conducted using a digestive-fluid mixture (DF) composed of artificial saliva (AS), fed-state simulated gastric fluid (FeSSGF), and artificial intestinal fluid (AIF). Because these fluids are nutrient-deficient and contain components that could adversely affect cells, Caco-2 tolerance to DF exposure was evaluated before conducting the PSNP experiments. Cells were seeded in complete EMEM at 2 × 10^4^ cells/cm^2^ in 24-well plates and maintained at 37 °C in a humidified 5% CO_2_ atmosphere until approximately 85% confluent, with medium changes as described above. Before cell exposure, DFs were heated at 90 °C for 5 min to inactivate enzymatic activity and then filtered through 0.22-μm polyethersulfone syringe filters (Genesee Scientific, San Diego, CA, USA; catalog no. 60-1850). The treated DF was applied either undiluted or at concentrations of 75%, 50%, or 25% (v/v) in complete EMEM. These digestive fluid–EMEM mixtures are hereafter referred to as DF/EM. Control wells received complete EMEM alone. Cell adherence, morphology, and monolayer integrity were evaluated by light microscopy at 2, 5, 9, and 24 h after exposure to identify the highest DF concentration that did not produce overt morphological evidence of cytotoxicity or monolayer disruption.

### 2.6 Rescue assay: Qi601 effect on Caco-2 surface-associated and intracellular PSNP

A post-exposure treatment assay was performed to determine whether Qi601 could reduce PSNP signal after PSNPs had already associated with Caco-2 cells (“rescue assay”). Caco-2 cells were cultured in 24-well plates in complete EMEM until approximately 85% confluent. On the day of the experiment, cells were washed twice with PBS and 1 mL/well of 0.1% PSNP v/v diluted in DF/EM was applied and incubated as before for 24 h at 37 °C.

After the initial 24-h PSNP exposure, the exposure medium was aspirated and cells were washed twice with PBS to remove unbound PSNPs. Qi601 was then applied at 3 mg/mL in DF/EM, and cells were incubated for an additional 5 or 24 h. Time-matched PSNP-only controls underwent the same aspiration and washing procedures but received DF/EM without Qi601 during the post-exposure incubation. Controls included untreated cells and cells exposed to Qi601 alone.

### 2.7 Recovery and quantification of PSNP fractions

Throughout this study, “surface-associated” refers to the extracellular PSNP fraction recovered from the cell surface, “intracellular” refers to the PSNP fluorescence recovered from the cell lysate fraction, and “cell-associated” refers collectively to the combined surface-associated and intracellular PSNP burden unless otherwise specified.

At each post-exposure time point, wash-recoverable surface-associated PSNPs and intracellular PSNPs from washed lysed samples were measured using a protocol adapted from Dos Santos et al., who established that three PBS washes were sufficient to remove non-internalized PSNPs before quantification of cell-associated PSNP fluorescence by flow cytometry and intracellular PSNP localization by confocal microscopy (dos Santos et al., 2011). Cells were washed three times with PBS to remove residual DF/EM, unbound Qi601, and loosely associated PSNPs. To recover the remaining surface-associated extracellular fraction, monolayers were incubated with 600 μL PBS containing Ca^2+^ and Mg^2+^ for 4 min at room temperature. The resulting surface-wash fraction was collected for fluorescence measurement.

Cells were subsequently lysed with prewarmed 0.5% Triton X-100 (Thermo Fisher Scientific, Waltham, MA, USA; catalog no. A16046.AE) in PBS. Lysates were centrifuged as before, and the resulting supernatants were collected for fluorescence and protein quantification. Total protein content per well was measured using the Pierce™ BCA Protein Assay Kit (Thermo Fisher Scientific, Waltham, MA, USA; catalog no. 23225). Replicate 200-μL aliquots of each sample were transferred to black, flat-bottom microplates, and fluorescence was measured as described above. Fluorescence values were background-corrected using the corresponding negative-control fractions and normalized to total protein content of the corresponding well.

Normalized surface-associated (SA) and intracellular (IC) PSNP signals were calculated as follows:


$$\begin{array}{l} SA=\frac{F_{SA\;sample}-F_{SA\;blank}}{TP}\\ IC=\frac{F_{CL\;sample}-F_{CL\;blank}}{TP} \end{array}$$

where *F*_*SA sample*_ is fluorescence measured in the surface-associated fraction, *F*_*SA blank*_ is fluorescence measured in the corresponding negative-control surface wash, *F*_*CL sample*_ is fluorescence measured in the cell lysate fraction, *F*_*CL blank*_ is fluorescence measured in the corresponding negative-control lysate, and *TP* is total cellular protein recovered from the corresponding well.

### 2.8 Protection assay: effect of Qi601 on Caco-2 PSNP association and uptake

Caco-2 cells were cultured in 24-well plates to approximately 85% confluence. On the day of the experiment, cells were washed twice with PBS and pretreated with Qi601 in 500 μL DF/EM for 30 min at 37 °C in a humidified atmosphere containing 5% CO_2_. The Qi601 concentration during preincubation was 6 mg/mL so that the final concentration after addition of the PSNP-containing medium was 3 mg/mL.

Following preincubation, 500 μL of DF/EM containing PSNPs at twice the intended final concentration was added to the appropriate wells, resulting in a final PSNP concentration of 0.1% (v/v) in a total volume of 1 mL per well. Cells were then incubated for 5 or 24 h at 37 °C as before. Controls included untreated cells, Qi601-only cells, PSNP-only cells, and cells treated with both Qi601 and PSNPs. Wash-recoverable surface-associated and intracellular PSNP fractions were collected and quantified as described above.

### 2.9 Transfer assay: PSNP transfer from Qi601-PSNP agglomerates to Caco-2 cells

Caco-2 cultures were used to determine whether PSNPs associated with Qi601–PSNP agglomerates (QPAs) after simulated digestion could subsequently become available for epithelial surface association or cellular uptake. Caco-2 cells were cultured in 24-well plates until they reached approximately 85% confluence.

Qi601 was suspended at 3 mg/mL in DF/EM and incubated with PSNPs at final concentrations of 0.1%, 0.5%, 1.0%, or 2.5% (v/v) for 1 h at 37 °C with orbital shaking at 250 rpm. Samples were then centrifuged at 1,700 × g for 10 min to sediment Qi601-associated material. The supernatant was collected, and its fluorescence was measured using a microplate reader to quantify PSNPs remaining outside the pellet and confirm QPA formation.

The Qi601-containing pellet was retained, washed three times with PBS, and resuspended in DF/EM to a final Qi601 concentration of 3 mg/mL. The amount of PSNP fluorescence associated with the washed QPA preparation immediately before Caco-2 exposure was measured directly and used as the denominator for calculation of apparent PSNP transfer. The QPA preparation was then applied to Caco-2 cultures for 5 or 24 h at 37 °C in a humidified atmosphere containing 5% CO_2_.

Dose-matched PSNP-only comparator conditions were prepared for each QPA condition. The amount of free PSNP added to each comparator well was matched to the measured PSNP fluorescence associated with the corresponding washed QPA preparation before cell exposure. These conditions were used to compare the cellular association of QPA-associated PSNPs with that of an equivalent amount of free PSNPs.

After 5 or 24 h of exposure, surface-associated and intracellular PSNP fractions were collected as described above. Background fluorescence was determined using the corresponding DF/EM and Qi601-only control fractions and was subtracted from each measurement.

Apparent PSNP transfer was calculated as the percentage of fluorescence recovered in the surface-associated and intracellular fractions relative to the fluorescence associated with the washed Qi601–PSNP agglomerate preparation immediately before Caco-2 exposure:


$$\begin{array}{l} PSNP\;Transf\;\left(\%\right)\\ =\frac{\left(F_{SA}-F_{SA\;BLANK}\right)+\left(F_{IC}-F_{IC\;BLANK}\right)}{F_{QPA}}\times 100 \end{array}$$

where *F*_*SA*_ is the fluorescence recovered from the surface-associated fraction, *F*_*SABLANK*_ is the fluorescence recovered from the corresponding membrane-bound negative control, *F*_*IC*_ is the fluorescence recovered from the intracellular fraction, *F*_*ICBLANK*_ is the fluorescence recovered from the corresponding intracellular blank control fraction, and *F*_*QPA*_ is the fluorescence associated with the washed QPA preparation before Caco-2 exposure.

PSNP transfer from QPAs to Caco-2 cells was calculated at each PSNP concentration, and results were reported as percentages relative to PSNP-only controls. Background fluorescence was determined from matched DF/EM and Qi601-only negative controls and subtracted before calculation. Measured signals remained within the instrument's validated linear range and were distinguishable from background.

To assess whether Qi601 altered Caco-2 membrane integrity and whether it affected PSNP-associated cytotoxicity, LDH release was measured in a separate 24-h experiment using Qi601 at 1, 3, or 5 mg/mL, with or without 1% (v/v) PSNPs. In the protection paradigm, cells were pretreated with Qi601 for 30 min before PSNP addition (Supplementary Figure 1).

### 2.10 Confocal microscopy imaging

Caco-2 cells were cultured in eight-chamber Millicell™ EZ Slide chamber slides (MilliporeSigma; Fisher Scientific, Waltham, MA, USA; catalog no. PEZGS0816) in complete EMEM to approximately 85% confluence. Cells were then exposed to 300 μL of DF/EM containing fluorescent PSNPs at a final concentration of 0.1% (v/v) and incubated for 24 h at 37°C in a humidified atmosphere containing 5% CO_2_. After exposure, the PSNP-containing medium was removed, and chambers were washed three times with PBS. Selected chambers then received 300 μL of Qi601 at 3 mg/mL in DF/EM for an additional 5 or 24 h. Controls included untreated cells, Qi601-only cells, and PSNP-only cells.

At each time point, chambers intended for quantitative imaging were washed three times with PBS to remove residual medium, loosely associated PSNPs, and non-adherent Qi601 material. One chamber exposed to both PSNPs and Qi601 received only a single PBS wash to retain a portion of the Qi601 material for qualitative visualization of Qi601–PSNP association at the epithelial surface. Images from this chamber were used only for qualitative assessment and were excluded from quantitative analysis.

Cells were fixed with 4% paraformaldehyde (Thermo Scientific, Thermo Fisher Scientific, Waltham, MA, USA; catalog no. AAJ60401AP) for 20 min at room temperature, washed three times with PBS and permeabilized with 0.1% Triton X-100 in PBS. F-actin was stained with rhodamine–phalloidin (Biotium, Fremont, CA, USA; catalog no. 00027) diluted 1:40 in PBS for 20 min at room temperature. Nuclei were stained with DAPI at 0.1 μg/mL in PBS for 10 min at room temperature (Biotium, Fremont, CA, USA; catalog no. 40043). Carbohydrate-rich material associated with Qi601 was visualized using CF™ 350-conjugated concanavalin A at 200 μg/mL for 15 min at 37°C (Biotium, Fremont, CA, USA; catalog no. 29137). DAPI and CF™ 350–ConA were not applied to the same chambers because of their overlapping fluorescence spectra. A deionized-water wash was performed between staining steps, and chambers were allowed to air-dry. ProLong™ Gold Antifade Mountant (Invitrogen, Thermo Fisher Scientific, Waltham, MA, USA; catalog no. P36930) was applied, coverslips were placed, and the mounting medium was allowed to cure overnight before imaging.

Images were acquired using laser-scanning confocal microscopy as before. DAPI and CF™ 350–ConA were excited using a 405 nm laser line with emission collected from 420–460 nm. Fluorescent PSNPs were excited using a 488 nm laser line with emission collected from 500–550 nm. Rhodamine–phalloidin was excited using a 543 nm laser line with emission collected from 565–625 nm. Signals corresponding to DAPI/CF™ 350–ConA, fluorescent PSNPs, and rhodamine–phalloidin were displayed in blue, green, and red, respectively. Identical acquisition settings, including laser power, detector gain, pinhole diameter, image dimensions, and z-step interval, were used for all quantitatively compared conditions. Image acquisition, ROI selection, and quantitative analysis were not blinded to treatment group.

For quantitative assessment, 14–20 ROIs per chamber were acquired from each of three independently treated chambers per condition. Z-stacks were acquired using identical imaging parameters across groups, and ImageJ version 1.54p (National Institutes of Health, MD, USA) was used for downstream PSNP fluorescence quantification. ROI measurements from each chamber were treated as technical subsamples and averaged to generate one value per chamber. The chamber was used as the experimental unit, with n = 3 chamber replicates per condition.

Each z-stack was converted using the extended depth of field ImageJ plugin and quantified within the segmented cellular region after background subtraction. A consistent fluorescence threshold was applied across all images to identify PSNP-positive pixels. Cellular PSNP fluorescence was calculated as the integrated fluorescence intensity of PSNP-positive pixels within each ROI. Integrated fluorescence was selected as the primary outcome because it accounts for both particle number and fluorescence intensity, whereas discrete particle counts do not capture variation in particle size or signal intensity.

### 2.11 Three-dimensional confocal localization analysis

Confocal z-stack imaging was used to assess the spatial localization of PSNP fluorescence after Qi601 treatment. Images were acquired using a spectral inverted laser-scanning confocal microscope (Fluoview FV1200, Olympus Corporation, Tokyo, Japan) and analyzed in Fiji (ImageJ; National Institutes of Health, Bethesda, MD, USA) using the 3D Object Counter plugin (Schindelin et al., 2012). For each region of interest (ROI), individual z-stack slices were converted to binary masks. Nuclear and cytoplasmic masks were combined to generate a three-dimensional cellular mask. PSNP fluorescence overlapping the three-dimensional cellular mask was classified as cell-localized, whereas fluorescence outside the cellular mask was classified as extracellular. PSNP object volumes across the complete z-stack were quantified using the 3D Object Counter plugin. A detailed step-by-step description of the three-dimensional image-processing and quantification workflow is provided in Supplementary Figure 2.

### 2.12 Scanning electron microscopy (SEM)

Caco-2 cells were seeded at 2 × 10^4^ cells/cm^2^ onto Transwell inserts with 0.4-μm pores (Costar, Corning Inc., Corning, NY, USA; catalog no. 3470) in 24-well plates. Complete EMEM was added to Transwell apical and basolateral compartments at volumes of 0.3 and 1.2 mL, respectively. Cultures were maintained at 37 °C as previously described. Digestates for SEM were generated using the sequential simulated digestion protocol described above. The apical compartment of Caco-2 cells at approximately 85% confluency were exposed to 300 μL of each digestate sample applied for 24 h. Following exposure, cultures were fixed overnight at 4 °C in 2.5% glutaraldehyde prepared in PBS. Samples were washed with PBS, dehydrated through a graded ethanol series and critical-point dried. The membranes were removed from the inserts, mounted to aluminum stubs, and sputter-coated with gold–palladium. For imaging Qi601 interactions with larger microplastics, Qi601 3 mg/mL was incubated with orbital shaking at 250 rpm with 10-μm polystyrene microspheres (1:100 v/v in PBS (Polysciences, Inc., Warrington, PA, USA; catalog no. 18140-2) for 1 h at 37 °C, deposited onto a polycarbonate filter and processed for SEM as above. Samples were imaged using a field-emission scanning electron microscope (SU5000, Hitachi High-Technologies, Tokyo, Japan). At least 15 representative fields of view were imaged per sample.

### 2.13 Atomic force microscopy (AFM)

A Qi601 stock suspension (OD600 = 4.0) was diluted 1:200 (v/v) in water and incubated with PSNPs diluted 1:1,000 (v/v) in water for 1 h. Controls consisted of PSNPs alone and Qi601 alone at the corresponding concentrations. Following incubation, the Qi601-containing samples were centrifuged at 1,700 × g for 10 min. The supernatants were discarded, and the resulting pellets were washed three times with PBS. Each washed pellet was resuspended in water to the original sample volume, and the resulting suspension was diluted 1:100,000 (v/v) in water. PSNP-only control samples were processed identically except that Qi601 was omitted. A 20-μL aliquot of each sample was deposited onto a clean, uncoated glass microscope slide and allowed to dry. Qi601-only, PSNP-only, and Qi601-plus-PSNP samples were imaged by atomic force microscopy (nGauge AFM, ICSPI Corp., Waterloo, ON, Canada).

### 2.14 Single-subject human proof-of-concept chewing gum study

To complement the *in vitro* PSNP-sequestration and intestinal epithelial cell-association assays, a qualitative chewing-gum experiment was conducted in one adult volunteer to determine whether Qi601 associates with microplastics in the human oral cavity under physiological conditions. The experiment was not designed to evaluate dose–response relationships, safety, capture efficiency, systemic absorption, or clinical outcomes. Chewing gum was selected as a reproducible and common source of oral microplastic exposure, releasing microplastic particles into human saliva during mastication (Lowe et al., 2025). A single volunteer participated in a minimal-risk chewing-gum and saliva-collection procedure conducted under ordinary food-use conditions and provided informed consent. Specimens were deidentified, no personal health information was collected, and no diagnostic, genetic, or clinical testing was performed. Institutional review board approval was not required for this proof-of-concept experiment.

Measures were taken to minimize exogenous plastic contamination during sample collection and processing. Glass serological pipettes, glass tubes, 100 mL glass beakers, and aluminum foil were used whenever feasible. Glassware and aluminum foil were rinsed three times with deionized water, submerged in 5% Extran^®^ 300 detergent solution (MilliporeSigma, Burlington, MA, USA, catalog no. 107555) for 45 min, and rinsed an additional three times with deionized water. The materials were then rinsed with 95% ethanol and air-dried under laminar airflow in a biosafety cabinet. After drying, the glassware was covered with pretreated aluminum foil. All liquid reagents used for saliva-sample processing were stored in decontaminated glassware. The participant was instructed to avoid eating, drinking, and oral-hygiene activities for at least 2 h before the experiment, to wear non-synthetic clothing, and to wash hands thoroughly before sample collection.

### 2.15 Saliva collection

A commercially available sugar-free chewing-gum pellet containing a synthetic gum base was used (Table 1). The brand was not identified because the experiment was not intended to evaluate or attribute the findings to a specific manufacturer or product. The same volunteer completed both the gum-only and gum-plus-Qi601 conditions sequentially, with the gum-only condition first.

**Table 1:** Chewing gum ingredients.

Ingredient category	Ingredients
Primary ingredients	Xylitol; gum base; maltitol syrup; mannitol
Contains 2% or less of	Natural and artificial flavor; maltodextrin; maltitol; lecithin (soy); artificial color including Blue 1 Lake and Blue 1; aspartame; acesulfame potassium; gum acacia; modified cornstarch; butylated hydroxytoluene (BHT); Neotame

Before sample collection, the volunteer was instructed not to swallow during each collection period and to expectorate saliva directly into the provided glass beaker as needed. To reduce baseline oral particulate contamination, the volunteer rinsed with 10 mL of sterile normal saline for 30 s and expectorated the rinsate into a glass beaker. This initial rinse fluid was retained. For the gum-only condition, the volunteer chewed one piece of gum and expectorated saliva until 10 mL had been collected, over approximately 5 min. As a washout prior to the subsequent gum-plus-Qi601 condition, another 10 mL saline oral rinse was performed to recover residual gum-derived particles from the oral cavity and added to the corresponding saliva sample.

For the gum-plus-Qi601 condition, the volunteer chewed a fresh piece of gum for approximately 5 s, after which 10 mg of Qi601 was introduced into the oral cavity. This brief initial chewing period was intended to permit mechanical release of gum-associated particles before concomitant exposure to Qi601 during continued mastication. The volunteer continued chewing and expectorating saliva for approximately 5 min until 10 mL had been collected. Another 10 ml saline oral rinse was performed and added to the corresponding saliva sample. Samples were stored at 4 °C until further processing.

### 2.16 Sample processing

Glass serological pipettes, glass tubes, and aluminum foil, cleaned as described above, were used throughout sample processing. Where feasible, samples were handled under laminar airflow to minimize airborne particulate contamination. During centrifugation, the glass tubes were nested within 15 mL conical tubes to fit the centrifuge rotor.

A procedural control underwent the same laboratory handling, processing, and ambient laboratory-air exposure as the experimental saliva samples and was used for background correction. An additional ambient-air control was collected to independently monitor airborne particle deposition during sample processing. Because ambient-air exposure was already incorporated into the procedural control, the ambient-air control was not used for additional background subtraction, thereby avoiding double correction for the same potential contamination source.

Saliva samples were transferred from the glass collection beakers to 25 mL glass tubes and allowed to sediment at room temperature for 30 min. The supernatant was carefully removed, and the sediment-enriched fraction containing gum-derived particles and residual biological material, as well as Qi601 in the gum-plus-Qi601 condition, was retained.

Enzymatic processing conditions were selected through preliminary method-optimization experiments to reduce salivary organic material while minimizing Qi601 detachment or degradation during sample preparation. Trypsin solution was prepared by diluting 0.25% trypsin containing 2.21 mM EDTA 1:1 with PBS (Corning, USA; catalog no. 25-053-CI). Four milliliters of prewarmed 0.125% trypsin solution were added to each sediment-containing tube. Samples were transferred to 5 mL glass tubes, covered with aluminum foil, and incubated at 37 °C for 45 min to reduce proteinaceous and cellular material. Samples were then centrifuged at 1,700 × g for 5 min, and the supernatants were discarded. The resulting pellets were washed once with PBS, centrifuged again at 1,700 × g for 5 min, and the wash supernatants were discarded.

DNase I treatment was then performed to reduce residual extracellular DNA (RNase-Free DNase Set; Qiagen, CA, USA; catalog no. 79254). DNase I was reconstituted according to the manufacturer's instructions, and 160 μL of the reconstituted enzyme solution was added to each pellet. Samples were incubated at room temperature for 15 min and centrifuged at 1,700 × g for 5 min, after which the supernatants were discarded. Pellets were washed with 500 μL of PBS, centrifuged as before, and wash supernatants discarded. Pellets retained for further analysis.

### 2.17 Saliva microscopic and spectroscopic analysis

#### 2.17.1 Fluorescence microscopy

Nile Red staining was used to visualize fluorescent hydrophobic polymer-like particles. Nile Red (Sigma-Aldrich, St. Louis, MO, USA; catalog no. 72485) was prepared in DMSO and diluted in PBS to 0.005% w/v. Pellets were resuspended in 200 μL stain, incubated for 5 min at room temperature, and centrifuged at 1,700 × g for 5 min. Residual dye was removed by sequential washes with 70% ethanol and 500 μL deionized water, each followed by centrifugation at 1,700 × g for 5 min. Pellets were resuspended in 1 mL deionized water, diluted 1:10, and 25 μL was mounted under a coverslip. Samples were examined using a standard fluorescence microscope (BX60, Olympus Corporation, Tokyo, Japan) with excitation and emission ranges of 540–550 nm and 572–620 nm, respectively. Nile Red-positive particles were considered presumptive gum-derived microplastics when their fluorescence colocalized with intrinsic UV fluorescence and/or exceeded the signal observed in baseline saliva controls. These findings were consistent with hydrophobic polymeric material but were not chemically specific for microplastics.

#### 2.17.2 Laser direct infrared (LDIR) spectroscopy

Laser direct infrared (LDIR) spectroscopy was then used for chemical characterization of particles released into saliva from chewing gum. Processed unstained saliva samples were deposited in 500-μL aliquots onto deposited onto low-emissivity infrared-reflective glass slides (Kevley Technologies, Chesterfield, OH, USA; catalog no. NC0733469), air-dried under laminar airflow, and analyzed by using an Agilent 8700 LDIR Chemical Imaging System (Agilent Technologies, Inc., Santa Clara, CA, USA). Entire sample automated particle analysis was performed using the instrument's microplastic-analysis workflow, which classifies particles by comparing their infrared spectra with reference-library spectra. Preliminary particle images were acquired at 1,442 cm^−1^ using a working particle-size range of 20–500 μm. Because the available LDIR reference library did not include spectra specific to chewing-gum-base elastomers, a full infrared spectrum was acquired for each particle and compared with the reference library using Clarity software, version 1.7.17 (Agilent Technologies, Inc., Santa Clara, CA, USA).

Because this proof-of-concept experiment evaluated the association of Qi601 with gum-base elastomers, the primary analysis focused on particles classified by LDIR as “Rubber.” Chewing-gum base contains synthetic polymers and elastomers, including styrene–butadiene rubber, isobutylene–isoprene copolymer, polyethylene, polyisobutylene, and polyvinyl acetate (U.S. Food and Drug Administration, 2026). Accordingly, particles assigned to the library category “Rubber” were considered the most relevant indicator of anthropogenic elastomeric particles consistent with gum-base origin. Spectral similarity was expressed as a hit quality index (HQI) ranging from 0 to 1, with higher values indicating greater similarity between the measured and reference spectra. Particles with HQI values ≥ 0.70 were included in the polymer-classification analysis, whereas assignments below this threshold were considered insufficiently reliable and excluded from subsequent analyses (Whiting et al., 2022). Other polymer assignments were treated as secondary observations because they could reflect oral, environmental, packaging, textile, or procedural contamination.

### 2.18 Time-lapse sedimentation assay

Red-dyed particles were selected to facilitate macroscopic visualization of sedimentation during time-lapse imaging. Qi601 (33 mg) was resuspended in 1 mL of PBS in a 15 mL conical tube. Red-dyed 0.20-μm polystyrene microspheres (100 μL; Polybead^®^ Polystyrene Red Dyed Microspheres, Polysciences, Warrington, PA, USA; catalog no. 15705) were then added from stock. The negative control contained the same quantity of microspheres in PBS without Qi601. Samples were incubated at 37 °C for 10 min with orbital shaking at 250 rpm, 10 mL of PBS added to each tube, and the tubes inverted several times to produce uniformly dispersed suspensions. The samples were left undisturbed at room temperature and recorded continuously for 5 min using an iPhone camera (iPhone 17, Apple Inc., Cupertino, CA, USA) at 1080p (HD) and 30 frames per second (fps) as Qi601–particle aggregates sedimented to the bottom of the tube. The negative-control suspension was recorded in parallel under the same conditions.

### 2.19 External analytical facilities

Confocal microscopy was performed by the authors using instrumentation at the University of South Florida Morsani College of Medicine. The authors retained responsibility for ROI selection, quantitative analysis, and interpretation of the confocal data. SEM was performed at the University of Florida Interdisciplinary Center for Biotechnology Research, where facility personnel operated the microscope, acquired images, selected fields and representative images, and performed imaging interpretation. LDIR analysis was performed at the Midwest Laboratory for Microplastic Research, Northern Illinois University. Saliva samples were prepared by the authors and submitted to the facility for analysis of the entire 0.5 mL samples. Particle detection and counting, polymer classification, and HQI assignment were performed automatically by the LDIR instrument/software without manual quantitative analysis or interpretation, and the resulting dataset was provided unedited to the authors for interpretation. All other experiments were performed in the authors' laboratory.

### 2.20 Statistical analysis

Statistical analyses were performed using Microsoft Excel 2021 (Version 2108; Microsoft Corporation, Redmond, WA, USA) with the Analysis ToolPak add-in. Data are presented as mean ± SD. Experimental units and true n are reported in each figure legend. Repeated measurements of the same sample and multiple ROIs obtained from the same well or chamber were treated as technical subsamples. Technical replicate measurements were averaged within each biological replicate, and the biological replicate was used as the experimental unit for statistical analysis. Comparisons between two independent groups were performed using unpaired, two-tailed Student's *t* tests. Comparisons involving multiple groups were analyzed using one-way ANOVA followed by Dunnett's multiple-comparisons test vs control. Effect-size estimates for the epithelial protection, post-exposure rescue, and transfer comparisons were expressed as mean differences between groups, with 95% confidence intervals calculated using Welch's method. For transfer percentages, mean transfer estimates and corresponding 95% confidence intervals were calculated using one-sample t-based intervals. For microscopy data, ROI-level measurements within each chamber were averaged to generate a single chamber-level value for statistical analysis. Statistical significance was defined as *p* < *0.05*. Significance levels are denoted as follows: *ns*, *p* ≥ 0.05, ^*^p < 0.05, ^**^p < 0.01, ^***^p < 0.001, ^****^p < 0.0001.

## 3 Results

Initial dose-response experiments showed a dose-dependent reduction in PSNP fluorescence remaining in the supernatant, reaching 87.6% at 3 mg relative to the PSNP-only control, with little additional reduction at higher doses (Figure 2A). We next evaluated whether this result was maintained in simulated digestive fluids. Qi601 reduced supernatant PSNP fluorescence in each digestive fluid tested, with a significant reduction detectable within 2 min in simulated salivary fluid. The reduction in PSNP fluorescence remaining in the supernatant varied among digestive conditions, reaching approximately 37% in simulated salivary fluid, 96% in simulated gastric fluid, 24% in simulated intestinal fluid, and 92% in the sequential salivary–gastric–intestinal digestion model. Each reduction was significant relative to its corresponding control without Qi601 (all *p* < *0.0001*; Figures 2B, C). These findings are consistent with substantial PSNP sequestration by Qi601, as earlier defined.

**Figure 2:**
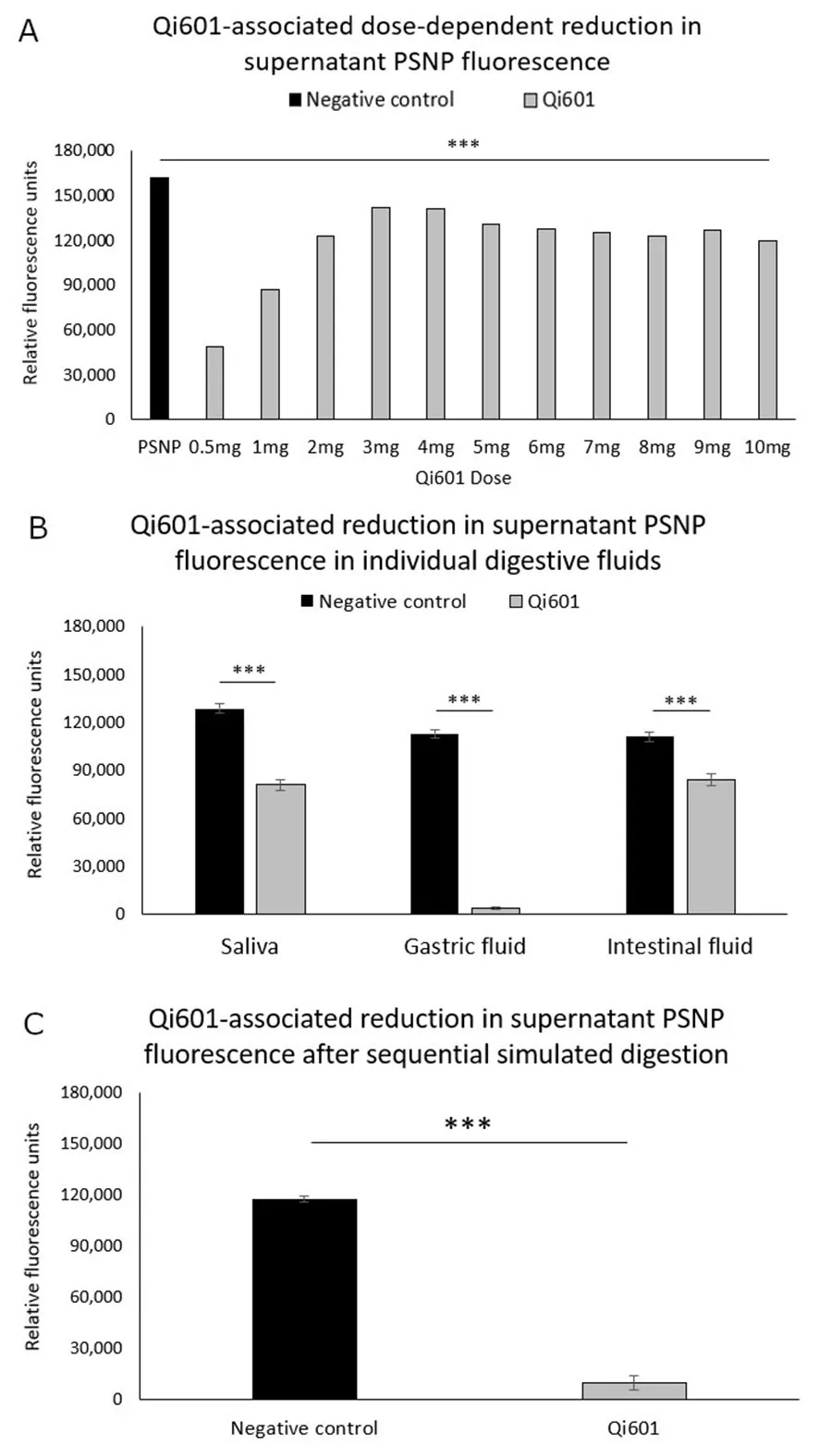
PSNP sequestration by Qi601 in simulated digestive fluids, assessed by supernatant fluorescence. **(A)** Dose-dependent PSNP sequestration by Qi601 in PBS, quantified as the reduction in PSNP fluorescence remaining in the supernatant (*n* = 3 biological replicates, each comprising 3 technical replicates). **(B)** PSNP fluorescence remaining in the supernatant after incubation with Qi601 (3 mg/mL) in individual simulated digestive fluids (*n* = 9 biological replicates, each comprising 3 technical replicates). **(C)** PSNP fluorescence remaining in the supernatant after incubation with Qi601 (3 mg/mL) in a sequential simulated digestive-fluid model of gastrointestinal transit (negative control *n* = 3 biological replicates; each comprising 3 technical replicates; Qi601 treatment group *n* = 9 biological replicates; each comprising 3 technical replicates). Reductions in supernatant PSNP fluorescence were calculated relative to the corresponding PSNP-only controls. Data are presented as mean ± SD. RFU, relative fluorescence units. *p < *0.05*, **p < *0.01*, ***p < *0.001*, ****p < *0.0001*.

To determine if simulated digestion conditions altered the ability of Qi601 to protect intestinal epithelial cells from PSNP exposure, Qi601 was subjected to sequential digestive-fluid exposure and then applied to Caco-2 monolayers maintained in digestive fluid diluted 1:2 in complete culture medium. We next evaluated three complementary epithelial exposure paradigms: protection, post-exposure rescue, and transfer. In the post-exposure (“rescue”) assay, Qi601 significantly reduced cell-associated PSNP fluorescence after exposure of epithelial monolayers to PSNPs for 24 h (Figure 3). This effect was time dependent based on duration of Qi601 treatment. After 5 h of Qi601 treatment, no significant reduction was observed in either surface-associated or intracellular fractions (Figures 3A, B). However, 24 h of Qi601 treatment significantly reduced both surface-associated PSNP by 77% (*p* = 0.0002) and intracellular PSNP by 43% (*p* = 0.0014; Figures 3C, D). In a separate LDH experiment using 1% PSNPs, LDH release in Qi601-alone conditions was lower than in untreated cells (*p* = 0.006). Furthermore, Qi601 significantly reduced LDH release in PSNP-exposed cells at all concentrations tested compared with PSNP treatment alone (*p* ≤ 0.011), consistent with Qi601 attenuation of cellular cytotoxicity associated with PSNP exposure (Supplementary Figure 1).

**Figure 3:**
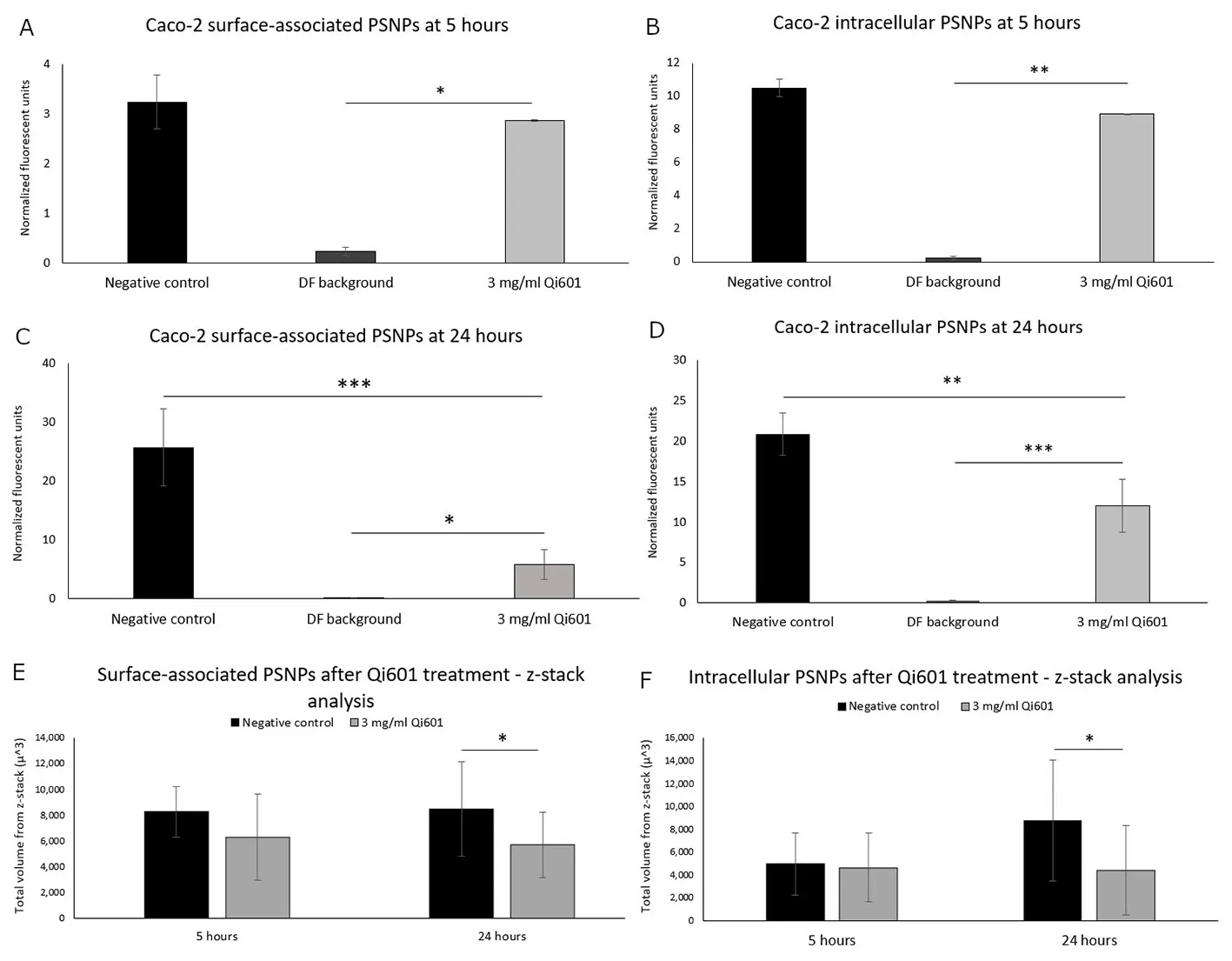
Post-exposure Qi601 reduces epithelial PSNP burden. Caco-2 monolayers were exposed to PSNPs for 24 h and treated with Qi601 at 3 mg/mL or control medium for 5 or 24 h. For fluorometric assays, fluorescence was normalized to the protein content of each well and expressed as RFU/μg protein. **(A)** Surface-associated PSNP fluorescence after 5 h of Qi601 treatment (*n* = *3* biological replicates, comprised of 3 technical replicates per group). **(B)** Intracellular PSNP fluorescence after 5 h of Qi601 treatment (*n* = *3* biological replicates, comprised of 3 technical replicates per group). **(C)** Surface-associated PSNP fluorescence after 24 h of Qi601 treatment (*n* = 5 biological replicates, comprised of 3 technical replicates per group). **(D)** Intracellular PSNP fluorescence after 24 h of Qi601 treatment (*n* = 5 biological replicates, comprised of 3 technical replicates per group). **(E)** Confocal z-stack analysis of surface-associated PSNP fluorescence after 5 and 24 h of Qi601 treatment (*n* = 3 biological replicates, comprised of 14 ROIs). **(F)** Confocal z-stack analysis of intracellular PSNP fluorescence after 5 and 24 h of Qi601 treatment (*n* = 3 biological replicates, comprised of 14 ROIs). Data are presented as mean ± SD. RFU, relative fluorescence units. *p < 0.05; **p < 0.01; ***p < 0.001.

Confocal microscopy was used to evaluate the localization of PSNP-associated fluorescence and to corroborate the fluorometric findings. Z-stack imaging was used to distinguish signal associated with the apical cell surface from signal located within the cellular volume. Consistent with the fluorometric assay, no significant reduction in either surface-associated or intracellular PSNP fluorescence was detected after 5 h of Qi601 treatment. After 24 h, both measures were significantly reduced, although the magnitude of the effect differed from that measured using the plate-based fluorescence assay. After 24 h, confocal z-stack analysis showed an approximately 33% reduction in surface-associated PSNP fluorescent volume and an approximately 50% reduction in intracellular PSNP fluorescent volume compared with PSNP-only controls (*p* = 0.028; Figure 3E; *p* = 0.035; Figure 3F). Confocal imaging therefore corroborated the plate-based findings by demonstrating reduced surface-associated and intracellular PSNP fluorescence, with the separate subcellular analysis showing that this effect included a significant reduction in perinuclear PSNP signal. These findings indicate that Qi601 reduced epithelial PSNP burden even after 24 h of prior PSNP exposure.

Prior studies in Caco-2 cells have shown that PSNP internalization is established at approximately 24 h (Liu Z. et al., 2025), localizing predominantly to perinuclear areas and cytoplasmic vesicular compartments including vacuoles and lysosomes (Domenech et al., 2021). We therefore used confocal laser-scanning microscopy to more closely examine the subcellular distribution of PSNP-associated fluorescence in Caco-2 cells exposed to PSNPs for 24 h before Qi601 treatment.

Consistent with rescue assay results, pixel-based quantitative confocal analysis demonstrated a Qi601-mediated reduction in intracellular PSNP signal in pre-exposed Caco-2 cells. After 24 h of Qi601 treatment, significant reductions were observed in whole-cell PSNP fluorescence (*p* = 0.0015; Figure 4A) and perinuclear PSNP fluorescence (*p* = 0.0018; Figure 4C) compared with control, whereas cytoplasmic PSNP-associated fluorescence showed a smaller, non-significant decrease (Figure 4B). These effects were not significant after 5 h of Qi601 treatment in any measured compartment (Figures 4A–C). Qi601 did not significantly reduce cytoplasmic PSNP signal at either time point (Figure 4B).

**Figure 4:**
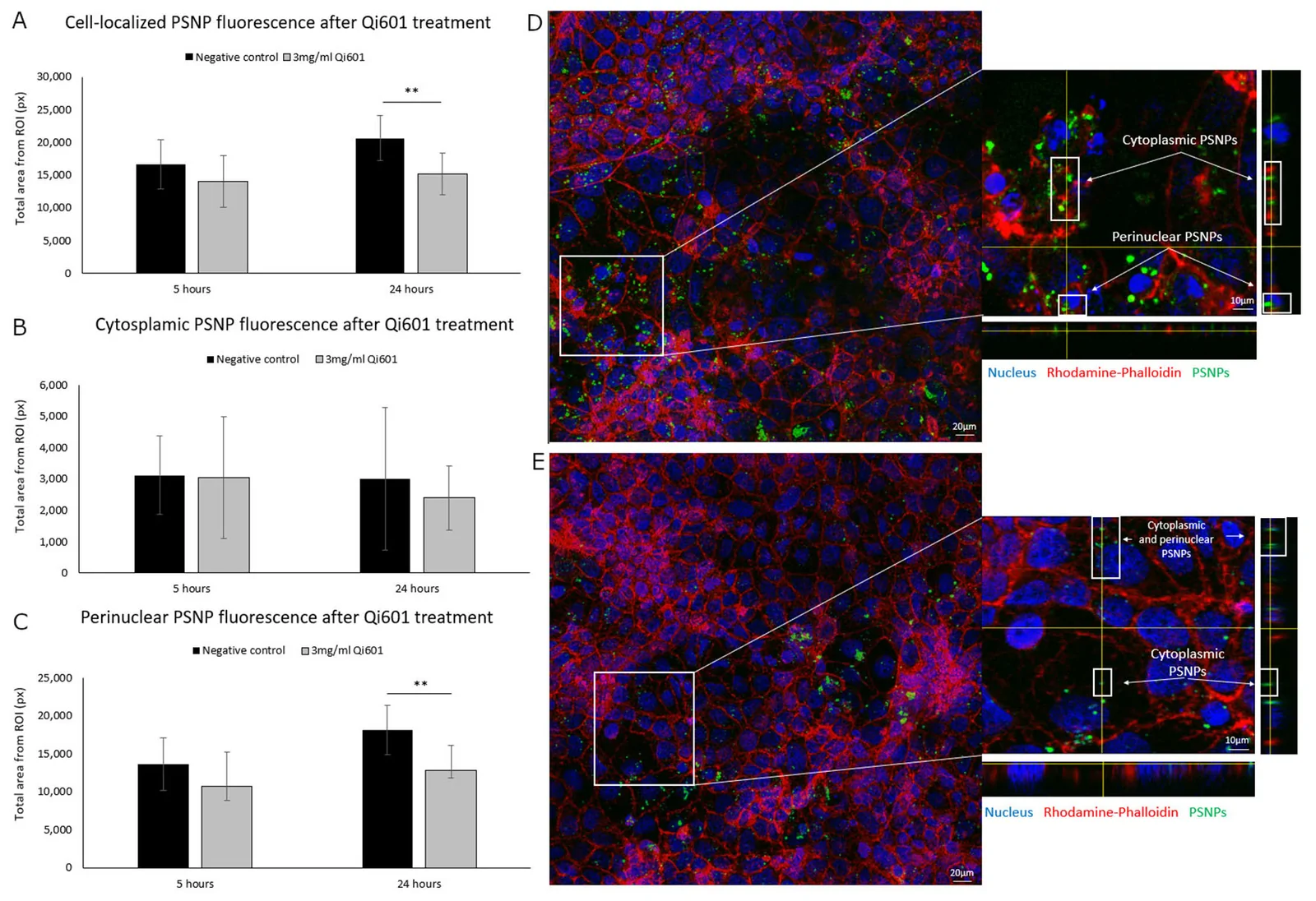
Post-exposure Qi601 treatment reduces cell-localized PSNP fluorescence. Cell-localized PSNP fluorescence was quantified within the entire cellular region of interest (ROI) **(A)**, cytoplasmic ROI **(B)**, and perinuclear ROI **(C)** at 5 h and 24 h after Qi601 treatment (*n* = *3* biological replicates; 14–20 ROI technical replicates analyzed per condition). Representative confocal images of Caco-2 monolayers exposed to PSNPs for 24 h without **(D)** or with **(E)** Qi601. Left panels show the analyzed fields; enlarged regions and orthogonal z-stack views are shown on the right. PSNPs are green, F-actin is red, and nuclei are blue. PSNP fluorescence was quantified as the integrated fluorescence intensity of PSNP-positive pixels within each ROI. Data are presented as mean ± SD. *p < 0.05, **p < 0.01, ***p < 0.001.

Representative CLSM images supported the quantitative findings. At 24 h, untreated control cells showed abundant PSNP fluorescence throughout the cell, with prominent perinuclear accumulation consistent with intracellular redistribution after uptake (Figure 4D). Qi601-treated cells showed an overall reduction in PSNP fluorescence, with markedly decreased perinuclear signal (Figure 4E). Taken together, the rescue assay and CLSM analysis indicate that Qi601 reduces cell-localized and perinuclear PSNP burden even after epithelial PSNP exposure.

Cellular protection assays were consistent with rapid-onset Qi601-mediated inhibition of PSNP-cellular association. At 5 h, Qi601-protected cells showed 65% lower surface-associated PSNP fluorescence (*p* < *0.0001*; Figure 5A) and 67% lower intracellular PSNP fluorescence (*p* < *0.0001*; Figure 5B) compared with control cells. The protective effect diminished but remained significant at 24 h, when Qi601-treated Caco-2 cells showed 41% lower surface-associated PSNP fluorescence (*p* = 0.004; Figure 5A) and 33% lower intracellular PSNP fluorescence (p = 0.021; Figure 5B) compared with controls. Compared with the rescue assay, the protection assay showed earlier reductions in both surface-associated and intracellular PSNP fluorescence, whereas significant rescue effects were observed only after 24 h of Qi601 treatment.

**Figure 5:**
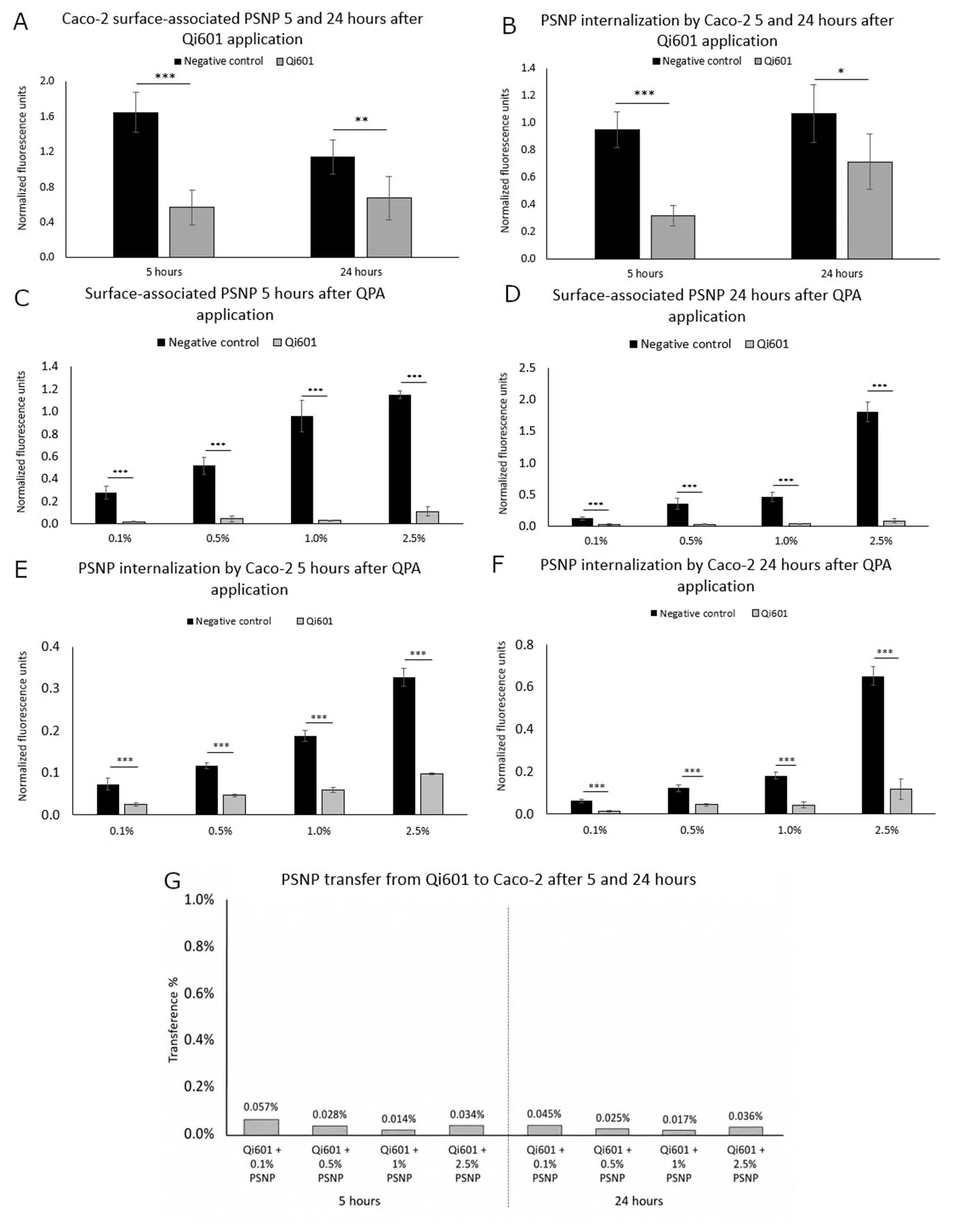
Qi601 reduces PSNP–epithelial association with minimal transfer of Qi601-sequestered PSNPs to cells. **(A, B)** Protection assay. Caco-2 monolayers were pretreated with Qi601 (3 mg/mL) before PSNP exposure. Surface-associated **(A)** and intracellular **(B)** PSNP fluorescence were measured at 5 and 24 h. **(C-F)** Transfer assay. Pre-formed Qi601-PSNP agglomerates (QPAs) were applied to Caco-2 monolayers. Surface-associated PSNP fluorescence was measured after 5 h **(C)** and 24 h **(D)**, and intracellular PSNP fluorescence after 5 h **(E)** and 24 h **(F)**, across PSNP concentrations of 0.1–2.5%, and compared with PSNP-only controls. **(G)** Percentage of Qi601-associated PSNP fluorescence transferred to Caco-2 monolayers at 5 and 24 h. Experiments included *n* = *3* biological replicates, each comprising 3 technical replicates. Fluorescence (RFU) was normalized to protein content per well. Data are presented as mean ± SD. RFU, relative fluorescence units. *p < 0.05, **p < 0.01, ***p < 0.001.

To evaluate the persistence of Qi601–PSNP association, we tested whether PSNPs pre-associated with Qi601 during sequential simulated digestion could transfer from Qi601–PSNP agglomerates (QPAs) to adjacent Caco-2 monolayers. Compared with PSNP-only controls, Qi601-associated agglomeration significantly reduced surface-associated (Figures 5C, D) and intracellular (Figures 5E, F) PSNP fluorescence at all concentrations and time points (all *p* < *0.0001*). Apparent PSNP transfer was minimal, ranging from 0.014%−0.057% at 5 h and 0.017%−0.045% at 24 h (Figure 5G). Effect-size estimates for the principal protection and post-exposure rescue comparisons, expressed as mean differences with corresponding 95% confidence intervals, together with mean transfer estimates and 95% confidence intervals, are provided in Supplementary Table 1. The Welch-based 95% confidence intervals were concordant with the significance pattern of the Student's *t*-test results. Together, these findings support persistent PSNP association with QPAs under the experimental conditions, with minimal transfer of PSNP fluorescence to epithelial cells.

Time-lapse imaging demonstrated that, under static conditions, Qi601 spontaneously promoted formation of visible PSNP agglomerates that progressively sedimented from suspension, whereas PSNPs in the absence of Qi601 remained uniformly dispersed (Supplementary Video 1). Complementary imaging modalities were then used to further characterize these Qi601–PSNP interactions. Fluorescence imaging showed dense PSNP signal colocalized with discrete multicellular Qi601 clusters (Figure 6A). AFM revealed PSNPs distributed along postbiotic surfaces in aggregated, organized topographies not observed in controls (Figure 6B). Confocal microscopy showed Qi601 clusters positioned above the epithelial monolayer, with PSNP signal predominantly localized to Qi601-associated material (Figure 6C). Orthogonal z-stack views showed comparatively little PSNP signal within the epithelial monolayer, supporting spatial separation between Qi601-associated PSNP agglomerates and the underlying cell layer. SEM demonstrated densely adherent PSNPs associated with mesh-like material on the surfaces of Qi601 agglomerates (Figure 6D), whereas in the absence of Qi601, PSNPs were observed adhering directly to Caco-2 cell microvilli (Figure 6E). Together, these findings showed close physical association between PSNPs and Qi601, consistent with the minimal epithelial transfer observed in the QPA assay.

**Figure 6:**
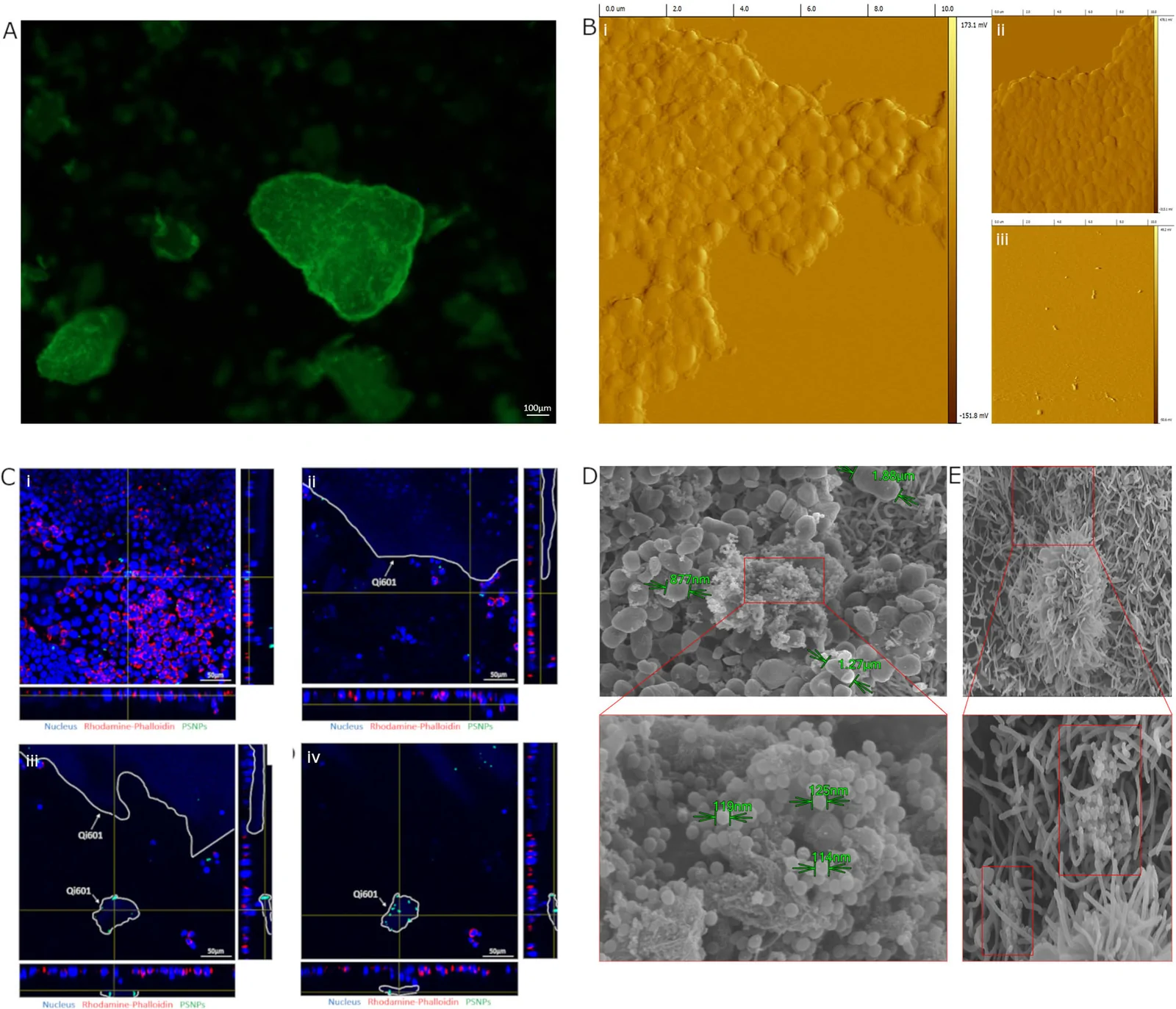
Multimodal imaging demonstrates extracellular association of PSNPs with Qi601 aggregates. **(A)** Fluorescence microscopy shows concentrated PSNP fluorescence in discrete Qi601 assemblies. **(B)** AFM phase images show PSNPs surrounding and on Qi601 surfaces (i), Qi601-only (ii) and PSNP-only controls (iii). **(C i-iv)** Confocal microscopy localizes Qi601-PSNP agglomerates (QPAs) above the Caco-2 monolayer. Caco-2 monolayers **(i)** show uptake of PSNPs. Qi601 aggregates **(ii, iii)** remain above the monolayer and accumulate PSNPs **(iv)**. Orthogonal z-stack views demonstrate separation between the aggregates (outlined in white) and the underlying cells, with multiple PSNP foci localized in smaller Qi601 assemblies. **(D)** SEM shows numerous PSNPs clustered on or within Qi601 aggregates on the Caco-2 epithelial surface. Higher magnification reveals PSNPs embedded in a mesh-like Qi601-associated matrix and closely apposed to Qi601 surfaces. **(E)** In the absence of Qi601, PSNPs adhere to Caco-2 cell microvilli. Scale bars are shown in each panel.

Having shown that Qi601 agglomerates PSNPs and limits epithelial PSNP exposure, we next examined whether this sequestration behavior could extend to more heterogeneous larger microplastics encountered *in vivo*. Recent studies have shown that mastication of chewing gum can release irregular polymeric fragments into saliva before swallowing (Lowe et al., 2025). Accordingly, chewing gum was selected as a proof-of-concept exposure model to qualitatively assess whether Qi601 could associate with gum-derived microplastics released during chewing.

Saliva samples collected after chewing gum with and without Qi601 demonstrated gum-derived microplastic particles in both conditions. Nile Red staining with UV excitation (Thammasanya et al., 2024) revealed fluorescent particles consistent with polymeric material under both conditions. These particles showed heterogeneous morphology and size distribution, with observed fragments ranging from approximately 20–100 μm, and occasional larger fragments extending to several hundred microns in this qualitative imaging model.

Qi601 appeared as variably sized multicellular structures and smaller postbiotic aggregates (Figure 7A). In Qi601-containing samples, postbiotic structures were observed in close association with gum-derived microplastic particles and, in some cases, appeared to bridge or aggregate multiple particles (Figure 7C). Composite fluorescence images showed colocalization between Qi601 material and plastic-associated signal (Figures 7C, D). The Qi601–plastic interface appeared broad and continuous rather than limited to isolated points of contact, consistent with extensive surface interaction between Qi601-associated material and the plastic fragments. A separate SEM model using 10-μm PSMPs similarly demonstrated microplastic particles adhering to and, in some fields, becoming embedded within Qi601 aggregates (Figure 7E). These findings indicated that Qi601 could physically associate with heterogeneous polymeric particulates generated during chewing and with larger defined microplastic particles.

**Figure 7:**
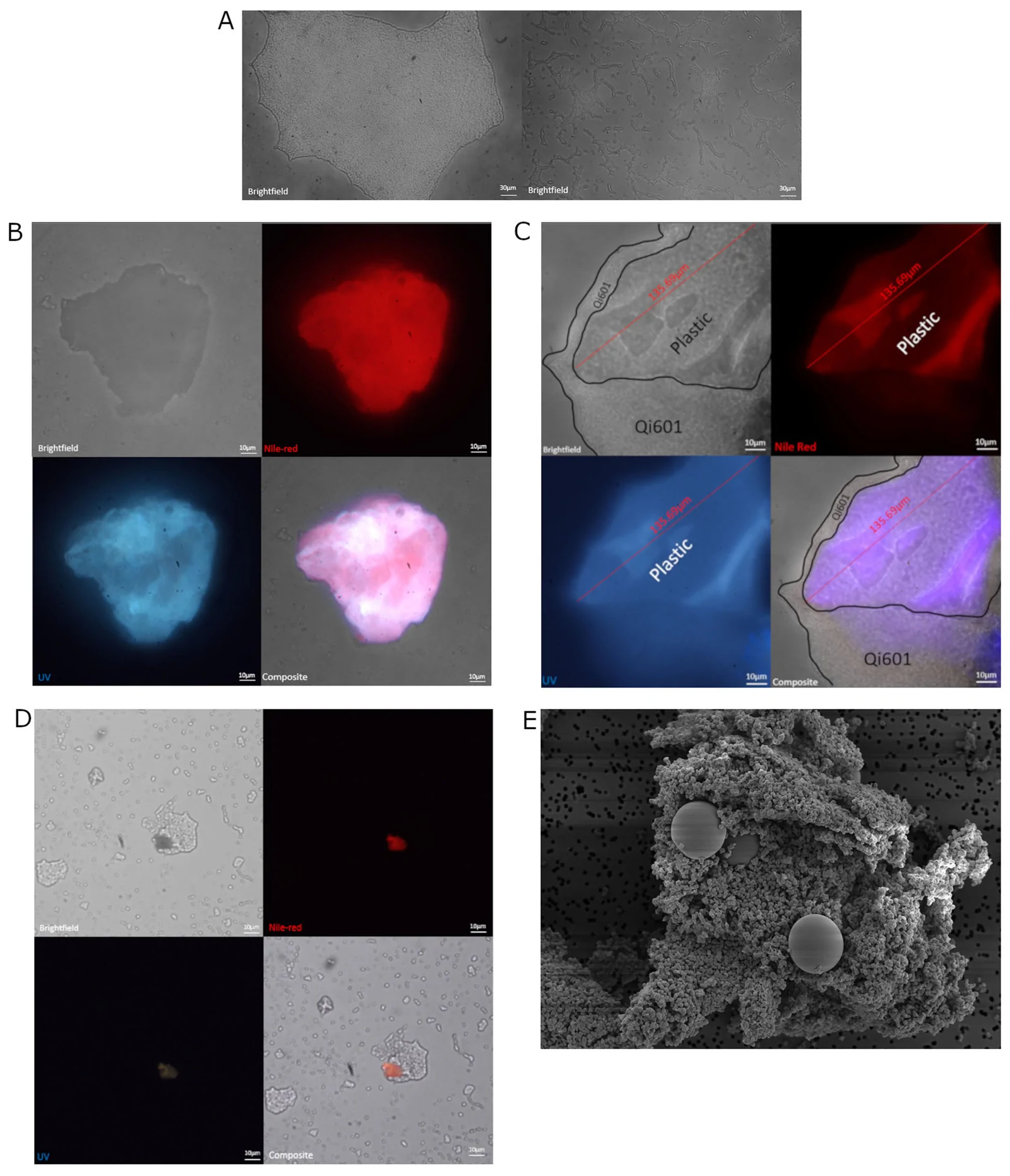
Microscopy of presumptive chewing gum-derived polymers and their association with Qi601. **(A)** Qi601 in PBS formed assemblies ranging from large aggregates **(left)** to smaller multicellular structures **(right)**. **(B)** Nile Red staining of saliva collected after gum chewing without Qi601 showed Nile Red-positive particles with fluorescence greater than surrounding biological debris. **(C, D)** Nile Red staining of saliva obtained after gum chewing with Qi601 showed Nile Red-positive particles within or closely associated with Qi601 aggregates. In **(C)**, the composite image delineates a polymer-like fragment partially surrounded by a Qi601 assembly. Grouped images **(B-D)** show brightfield, Nile Red fluorescence, UV fluorescence, and composite views. Images are representative of multiple ROIs examined per condition. **(E)** SEM image from a separate model showing a 10-μm PSMP embedded in a Qi601 aggregate. Scale bars are shown where indicated.

To chemically identify salivary Nile Red-staining particles, samples were subsequently analyzed with LDIR. Gum base is the insoluble component of gum responsible for its rubbery texture. United States food regulations permit certain synthetic polymers to be used in gum base: butadiene-styrene rubber, isobutylene-isoprene copolymer (butyl rubber) and polyisobutylene, as well as the non-elastomeric plastics polyethylene (PE), polyvinyl acetate (PVA) and certain vinyl co-polymers (U.S. Food and Drug Administration, 2026). Gum base of soft chew gum of the type used in this study often omits PVA and PE (Reggio et al., 1983). Accordingly, rubber particles were selected as the primary endpoint.

Synthetic rubber particles accounted for 81% of all microplastics identified by LDIR in saliva obtained during gum chewing, with or without Qi601 (Figure 8). Other polymer assignments were considered secondary and were not used in subsequent analysis. The experimental workflow is summarized in Figure 8A. The Qi601-only control contained no particles identified as rubber (Figure 8B). The number of recovered rubber-class particles in the gum-plus-Qi601 saliva sample was 43% lower than in the gum-only saliva sample (Figure 8B; 2,562 vs. 4,529 particles). After subtraction of the procedural-control rubber particle count, the background-adjusted recovered rubber particle counts were approximately 91% lower (Figure 8C; 2,152 vs. 185 particles). Representative LDIR particle maps showed abundant rubber particles in the gum-only saliva sample (Figure 8D), whereas the gum-plus-Qi601 saliva sample demonstrated markedly fewer detectable rubber particles (Figure 8E). Complementary Nile Red and UV imaging showed fluorescent gum-derived material in the gum-only saliva sample (Figure 8F) and markedly reduced fluorescence in the gum-plus-Qi601 sample (Figure 8G) with minimal background signal in the Qi601-only control (Figure 8H).

**Figure 8:**
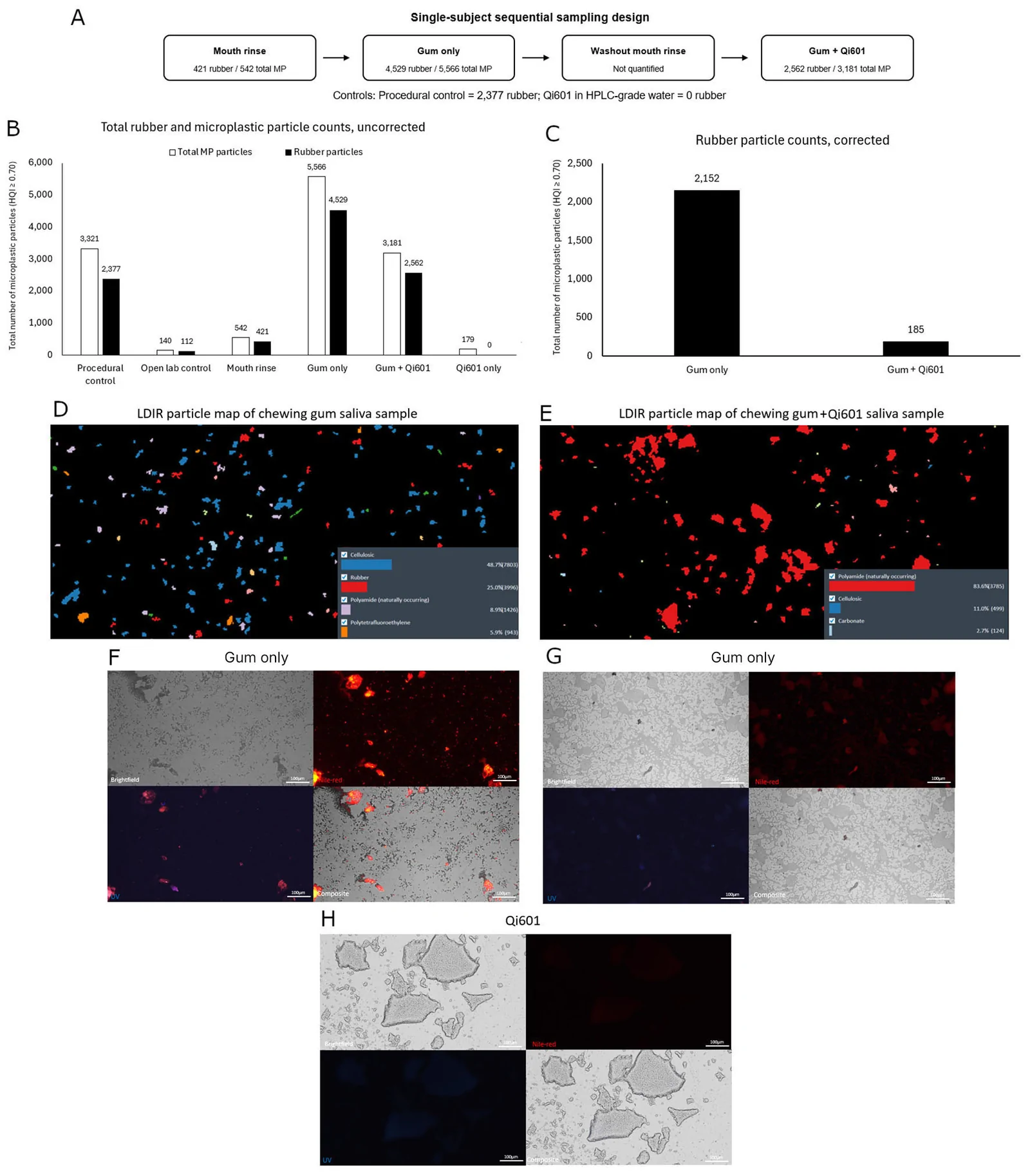
LDIR quantification of rubber particles after gum chewing with and without Qi601 in a single-subject experiment. **(A)** Experimental sequence: baseline mouth rinse, gum chewing, saliva collection, washout rinse, gum chewing with Qi601, and repeat saliva collection. Rubber and total microplastic particles were identified by LDIR using a HQI ≥ 0.70. Controls included a procedural control processed alongside the saliva samples and Qi601 in HPLC-grade water. **(B)** Uncorrected total microplastic and rubber particle counts. **(C)** Rubber particle counts after subtraction of the procedural control count. **(D, E)** Representative regions of interest (ROIs) from LDIR particle scans of saliva samples obtained after gum chewing without Qi601 **(D)** and with Qi601 **(E)**. **(F, G)** Representative brightfield, Nile Red fluorescence, UV fluorescence, and composite images of material recovered after gum chewing without Qi601 **(F)** and with Qi601 **(G)**. **(H)** Corresponding imaging of the Qi601-only control.

Taken together, the exploratory chewing-gum experiment, *in vitro* studies, and multimodal imaging support the conclusion that Qi601 can physically associate with nanoscale and microscale plastic particles across multiple experimental models.

## 4 Discussion

The present findings support a physical postbiotic sequestration model in which Qi601 associates with micro- and nanoplastics in the oral cavity and during simulated digestion, thereby reducing the fraction available for epithelial contact and uptake (Figure 9). This model is supported by convergent evidence from simulated digestion, epithelial protection and rescue assays, particle-transfer experiments, multimodal imaging, and an exploratory human proof-of-concept study.

**Figure 9:**
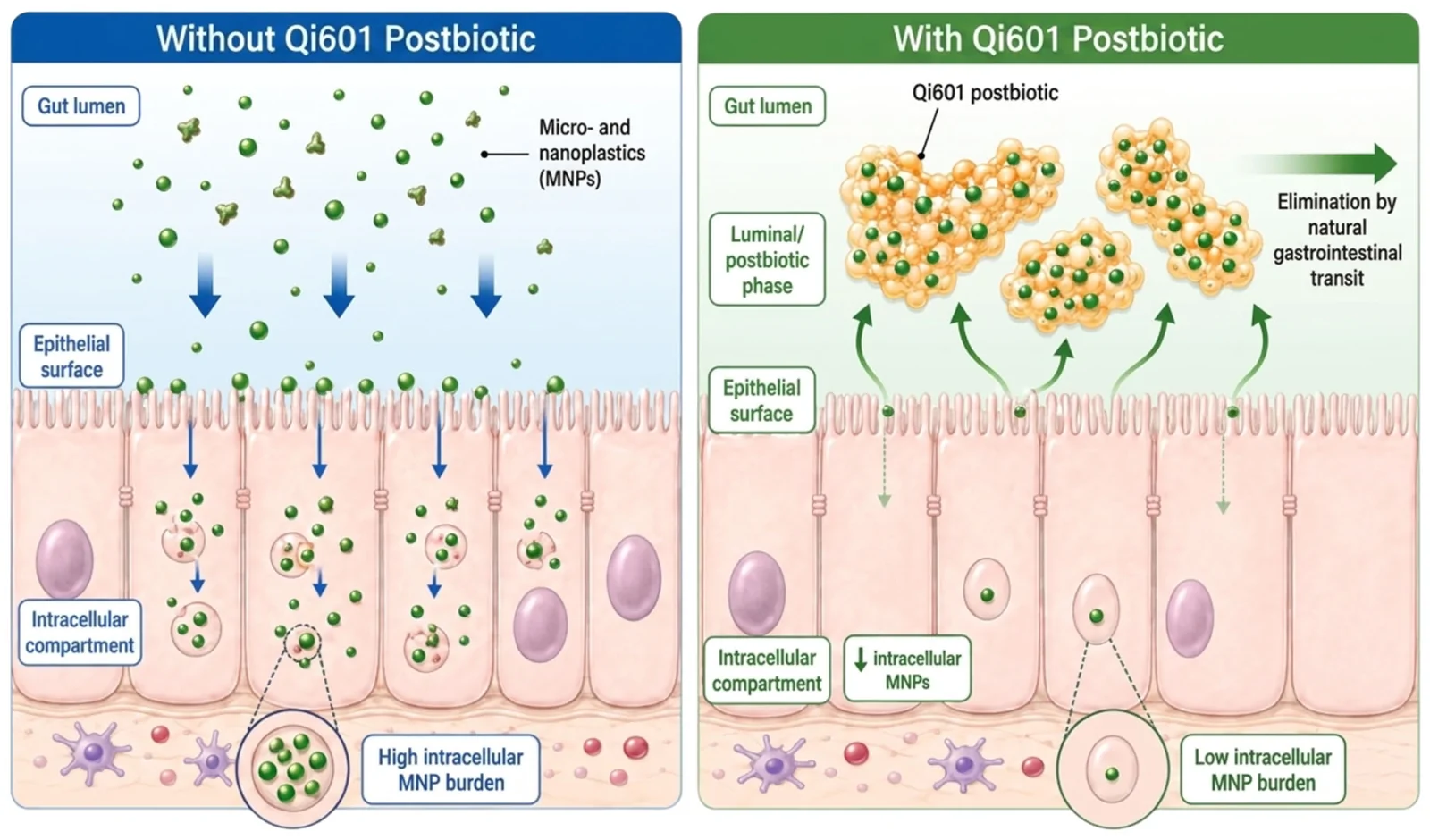
Proposed Qi601 postbiotic sequestration model. Qi601 agglomerates with ingested MNPs, thereby reducing epithelial surface association and intracellular particle burden. Retention of MNPs in the luminal compartment may facilitate their elimination through normal gastrointestinal transit. Created with BioRender.com.

The protection and post-exposure paradigms evaluated different stages of epithelial-particle interaction. Whereas Qi601 could rapidly sequester particles before epithelial uptake in the protection assay, the rescue assay examined its effects after a period during which PSNPs have been reported to localize to perinuclear and vesicular compartments (Domenech et al., 2021; Liu Y. Y. et al., 2025). These complementary approaches suggest that postbiotic sequestration may reduce epithelial particle burden both before and after cellular exposure. Qi601 also attenuated PSNP-associated LDH release from Caco-2 monolayers, providing independent evidence consistent with protection against PSNP-associated cytotoxicity.

To our knowledge, this is the first study to evaluate whether extracellular sequestration can reduce a previously established epithelial nanoplastic burden. Prior work in this regard appears limited to a single study using cell-permeable pharmacologic inhibition of intracellular transport to promote PSNP exocytosis (Han and Ryu, 2022). In contrast, the present findings suggest that extracellular sequestration may also reduce intracellular nanoplastic burden despite lacking direct intracellular activity. We are not aware of any prior studies evaluating whether a microbial product, live or inactivated, can produce a similar effect.

How an extracellular sequestrant reduced intracellular nanoplastic burden remains unknown. Because internalized particles are poorly accessible to extracellular Qi601, direct extraction is unlikely. Although not investigated here, possible mechanisms include intracellular vesicular recycling or exocytosis coupled with ongoing extracellular sequestration of released particles (Liu et al., 2021, 2023; Han and Ryu, 2022; Liu Y. Y. et al., 2025).

The transfer assay, time-lapse imaging and multimodal microscopy support the postbiotic sequestration model. Time-lapse imaging demonstrated the spontaneous formation and sedimentation of readily visible Qi601-PSNP agglomerates under static conditions, whereas PSNPs alone remained freely dispersed. These findings are consistent with Qi601-associated reduction in supernatant PSNP fluorescence observed under simulated digestive conditions in the adapted INFOGEST experiments. Qi601–PSNP complexes remained intact after the 24-h epithelial exposure experiments and subsequent confocal microscopy and SEM processing, supporting persistent postbiotic-particle association. Different incubation times, agitation parameters or gastrointestinal conditions could influence these findings. Collectively, complementary and orthogonal lines of evidence converge on the conclusion that Qi601 redistributes free PSNPs into postbiotic-associated agglomerates, resulting in limited epithelial transfer under the experimental conditions.

There is increasing interest in strategies that reduce human exposure to these foreign particles (Bora et al., 2024; Sofield et al., 2024). Most plastic-remediation approaches have focused on environmental removal or degradation but methods to limit gastrointestinal absorption of ingested MPs are beginning to receive attention (Teng et al., 2024; Demarquoy, 2025). Probiotic organisms have recently been explored as potential MNP biosorbents. Teng et al. demonstrated that selected live probiotic strains adsorb microplastics and enhance their excretion *in vivo* in mice (Teng et al., 2024). Shi et al. further showed that LAB-mediated effects on MNP toxicity depends not only on binding capacity but also on restoration of the gut environment (Shi et al., 2025). Zhao et al. demonstrated that live *L. plantarum* ZP-6 reduced PS-NP tissue retention, increased fecal excretion, and ameliorated inflammatory, barrier, and metabolic injury in murine models (Zhao et al., 2025). Whereas previous probiotic studies primarily evaluated downstream biological consequences of microbial–plastic interactions, the present study focused on luminal interception of particles before epithelial contact. Collectively, these studies and the present findings support microbial sequestration of MNPs as a promising strategy to reduce gastrointestinal MNP exposure, although the reported sequestration efficiencies and biological outcomes are not directly comparable because of substantial methodological differences.

In contrast to these probiotic studies, the present study evaluates a non-viable, biofilm-derived preparation as a physical MNP-sequestration platform. Extending prior probiotic studies of microbial–plastic association, Qi601 substantially reduced PSNP fluorescence remaining in the supernatant under simulated digestive conditions, consistent with particle sequestration, while reducing epithelial particle burden. Rather than demonstrating superiority over live probiotic approaches, the present findings show that bacterial viability is not required for physical association and agglomeration with MNPs. Previous studies of multiple probiotic strains showed loss of MP-binding activity after heat-inactivation (Lee et al., 2026). The preserved capacity of Qi601 to physically associate and agglomerate with MPs may relate to its production as a pre-formed biofilm rather than by heat-inactivation of planktonic cells. Biofilm-associated extracellular material provides an organized surface matrix capable of binding hydrophobic particles, consistent with the formation of the “plastisphere,” the microbial biofilms that colonize environmental plastic surfaces (Zettler et al., 2013).

Soluble cell-free components from heat-treated *L. delbrueckii* subsp. *bulgaricus* 2038 and *S. thermophilus* 1131 have previously been shown to suppress PSNP internalization in Caco-2 monolayers (Kobayashi et al., 2026). Notably, that study found no evidence of direct bacterial-PSNP aggregation. The present results extend those observations by supporting a distinct postbiotic sequestration model, whereby a non-viable postbiotic directly associates with PSNPs under simulated digestive conditions, reducing their availability for cellular interaction and uptake.

A postbiotic may offer a more controllable platform for luminal sequestration than a live probiotic (Salminen et al., 2021). Because microbial sequestration of plastic particles likely depends on physical surface interactions, Qi601 may serve as a physical scaffold for particle sequestration (Zhao et al., 2023). As a non-viable postbiotic, Qi601 could function as a transient luminal sequestrant, avoiding limitations associated with live probiotics, including viability loss, transfer of antimicrobial-resistance elements, unpredictable host colonization, and toxicity of plastic particles to the administered probiotic (Ma et al., 2023).

A non-viable MNP sequestrant may behave differently from a live organism at the epithelial surface. A live organism capable of retaining microplastics that also adheres to the intestinal epithelium could theoretically concentrate MNPs and their associated chemicals near the mucosa (Tumwesigye et al., 2023; Van Tassell and Miller, 2011). The biological consequences of MNP sequestration may therefore differ between live probiotics and non-viable postbiotics. As a transient sequestrant, a non-colonizing postbiotic designed to agglomerate MNPs while remaining predominantly within the lumen could offer theoretical advantages.

To our knowledge, this represents the first human *in vivo* proof-of-concept study directly demonstrating physical association between an administered intervention designed to capture microplastics and microplastic particles. The convergent evidence presented in this study supports the feasibility of intentional *in vivo* microplastic capture by an administered intervention in humans. These findings support postbiotic sequestration as a potential strategy to intercept dietary micro- and nanoplastics before epithelial interaction.

Several limitations should be considered when interpreting these findings. Most experiments used fluorescent polystyrene nanoplastic particles at concentrations selected to ensure reliable detection. While these model particles enabled controlled studies, they do not capture the heterogeneity of polymer composition, chemistry, morphology, size, or typical exposure levels characteristic of real-world dietary MNPs. This limitation was partially addressed by the chewing-gum study, which evaluated heterogeneous microplastic particles released from chewing gum. Additionally, omission of pancreatin from the adapted INFOGEST protocol limits physiological completeness of the simulated intestinal phase. Future studies should determine how pancreatic enzymes influence protein-corona formation and Qi601–MNP interactions.

Caco-2 cultures were not evaluated as barrier-competent monolayers. Accordingly, the findings support reduced epithelial nanoparticle burden but not epithelial barrier function. The washing and lysis procedures used to distinguish surface-associated from intracellular PSNP fluorescence may not have completely separated these compartments. This limitation was partially mitigated by orthogonal confocal z-stack analysis, which supported the localization findings. Confocal imaging and analysis were not blinded to treatment group, although standardized acquisition and analysis procedures were applied across groups. Additionally, the experiments reported here were performed using a single commercial-scale production lot of Qi601.

Finally, the chewing-gum experiment was designed as a single-subject, exploratory, proof-of-concept study intended to establish feasibility and therefore was not designed to assess dose response, safety, biodistribution, gastrointestinal elimination, or clinical efficacy. Although procedural and ambient-air controls were incorporated to assess background contamination, environmental contamination cannot be completely excluded in microplastic analyses. In addition, LDIR cannot unequivocally distinguish all chemically similar elastomeric polymers due to overlapping infrared spectra. However, because the same chewing-gum product was used in both experimental conditions, this limitation is unlikely to account for the observed between-condition differences.

Future *in vitro* studies should define the molecular basis of Qi601–MNP interactions, use barrier-competent intestinal models to evaluate epithelial barrier function and the biological consequences of epithelial MNP uptake, and assess postbiotic sequestration and dose-response relationships across a broader range of dietary MNPs under dynamic gastrointestinal conditions. Future human studies should characterize the gastrointestinal fate of Qi601–MNP agglomerates *in vivo*, including luminal retention and elimination, and validate these findings in larger studies.

Collectively, the present findings support a model in which Qi601 functions as a gastrointestinal platform for sequestering dietary micro- and nanoplastics (Figure 9). The findings reported here demonstrate rapid physical association and agglomeration of Qi601 with micro- and nanoplastics, reducing the pool of freely available particles for epithelial interaction and cellular uptake. Our model proposes that Qi601 forms MNP-containing agglomerates within the gastrointestinal lumen, which may subsequently undergo elimination during normal intestinal transit.

## Data Availability

The raw data supporting the conclusions of this article will be made available by the authors, without undue reservation.
