# Supplementary material for: Postbiotic sequestration of micro- and nanoplastics: in vitro intestinal epithelial protection and proof of concept in the human mouth
Source: Front Microbiol. 2026 Sep 21;17:1881786. doi: 10.3389/fmicb.2026.1881786 (PMC13635293; doi:10.3389/fmicb.2026.1881786)
Supplement: Supplementary file 1 [file Supplementary_file_1.docx]

**Supplementary Material**

**Supplementary Figure S1. Evaluation of Qi601 and PSNP effects on Caco-2 cytotoxicity. (A)** LDH release after 24 h exposure to Qi601 (1, 3, or 5 mg/mL), PSNPs (1%), or indicated controls. **(B)** LDH release expressed relative to the maximum-release (lysis) control. **(C)** LDH release after 30 min Qi601 pretreatment followed by PSNP exposure, compared with PSNP alone. **(D)** Cytotoxicity in Qi601-pretreated, PSNP-exposed cells expressed as a percentage of the PSNP-only condition. Qi601 alone did not increase LDH release relative to untreated controls, while Qi601 pretreatment significantly reduced LDH release compared with PSNP alone at all concentrations tested. Data represent *n* = 3 biological replicates, each with 3 technical replicates, and are presented as mean ± SD. *ns, p* ≥ 0.05; **p* < 0.05; *p* < 0.01; **p* < 0.001; *****p* < 0.0001.

**Method.** Caco-2 cells were cultured in 96-well plates in complete EMEM to approximately 85% confluence, washed twice with PBS, and exposed for 24 h to Qi601 (1, 3, or 5 mg/mL), PSNPs (1%), or both in DF/EM (1:2, v/v) at 37°C and 5% CO₂. For protection experiments, Qi601 was applied 30 min before PSNPs; PSNP-only cells served as the comparator. LDH release was measured using the CyQUANT™ LDH Cytotoxicity Assay Kit (Invitrogen, Thermo Fisher Scientific, Waltham, MA, USA; C20301). Untreated cells and the kit maximum-release condition served as spontaneous-release and positive lysis controls, respectively. Assay-background values were subtracted before calculation. Percent cytotoxicity was calculated as: % Cytotoxicity = [(experimental LDH release – spontaneous LDH release) / (maximum LDH release – spontaneous LDH release)] x 100. For the protection assay, cytotoxicity in each Qi601 + PSNP condition was expressed relative to the PSNP-only condition as: % of PSNP-only cytotoxicity = (cytotoxicity in Qi601 + PSNP condition/cytotoxicity in PSNP-only condition) × 100.

**Supplementary Figure S2. Three-dimensional confocal image analysis workflow.** **(A)** Three-dimensional rendering of a z-stack produced using the **3D Viewer** plugin. **(B)** Z-stacks were separated into individual slices using the **Stack to Images** command. Individual slices contained either cell-localized or extracellular PSNP fluorescence. **(C)** Each slice was split into the green channel (PSNPs), blue channel (nuclei), and red channel (F-actin). The blue and red channels were then merged to generate the three-dimensional cellular mask. **(D)** Images were converted to binary using the **Convert to Mask** command. The binary PSNP image represented the complete PSNP population (white foci), whereas the merged nuclear–F-actin image represented the three-dimensional cellular mask (larger white areas). **(E)** The three-dimensional cellular mask was subtracted from the complete PSNP image for each slice using the **Image Calculator** function to distinguish cell-localized from extracellular PSNP fluorescence. **(F)** The resulting image contained only extracellular PSNP fluorescence that did not overlap with the three-dimensional cellular mask. **(G)** Cell-localized and extracellular PSNP burdens were quantified across the entire z-stack using the **3D Object Counter V2** plugin, and PSNP volume (µm³) was calculated for each z-stack.

**Supplementary Video S1:** **Time-lapse recording of spontaneous Qi601–PSNP agglomeration and sedimentation.** Qi601-PSNP agglomerates rapidly sediment to the bottom of a 15-mL conical tube within 5 min (right), whereas PSNPs alone remain uniformly suspended in the absence of Qi601 (left). The video illustrates the dynamic and spontaneous formation of readily visible Qi601–PSNP agglomerates under static conditions. Qi601 (33 mg) was resuspended in 1 mL PBS in a 15-mL conical tube. Red-dyed 0.20-µm polystyrene microspheres (100 µL; Polybead® Polystyrene Red Dyed Microspheres, Polysciences, Warrington, PA, USA; catalog no. 15705) were added from stock. The negative control contained an identical concentration of microspheres in PBS without Qi601. Samples were incubated at 37°C for 10 min with orbital shaking (250 rpm), diluted with 10 mL PBS, and inverted several times to produce uniformly dispersed suspensions. Tubes were then left undisturbed at room temperature and recorded continuously for 5 min using an iPhone 17 (Apple Inc., Cupertino, CA, USA) at 1080p resolution and 30 frames/s. The negative control was recorded in parallel under identical conditions.

**Supplementary Table S1: Effect-size estimates and 95% confidence intervals for the epithelial protection, post-exposure rescue, and transfer experiments.** Mean differences between groups and corresponding 95% confidence intervals, calculated using Welch’s method, are reported for the protection and post-exposure rescue experiments. For the transfer experiment (Fig. 5G), the mean transfer estimate and corresponding 95% confidence interval were calculated using a one-sample *t*-based interval. Independent biological replicates were used as the experimental unit, with technical replicates averaged within each biological replicate.
